# Effect of Immobilisation on Neuromuscular Function In Vivo in Humans: A Systematic Review

**DOI:** 10.1007/s40279-019-01088-8

**Published:** 2019-03-21

**Authors:** Matthew Campbell, Jo Varley-Campbell, Jon Fulford, Bryan Taylor, Katya N. Mileva, Joanna L. Bowtell

**Affiliations:** 1 School of Sport and Health Science, University of Exeter, St Lukes Campus, Exeter, EX1 2LU UK; 2 Department of Clinical, Educational and Health Psychology, Centre for Outcomes Research and Effectiveness (CORE), University College London, London, WC1E 7HB UK; 3 University of Exeter Medical School, University of Exeter, St Lukes Campus, Exeter, EX1 2LU UK; 4 School of Biomedical Sciences, University of Leeds, Leeds, LS2 9JT UK; 5 Sport and Exercise Science Research Centre, London South Bank University, London, SE1 0AA UK

## Abstract

**Background:**

Muscle strength loss following immobilisation has been predominantly attributed to rapid muscle atrophy. However, this cannot fully explain the magnitude of muscle strength loss, so changes in neuromuscular function (NMF) may be involved.

**Objectives:**

We systematically reviewed literature that quantified changes in muscle strength, size and NMF following periods of limb immobilisation in vivo in humans.

**Methods:**

Studies were identified following systematic searches, assessed for inclusion, data extracted and quality appraised by two reviewers. Data were tabulated and reported narratively.

**Results:**

Forty eligible studies were included, 22 immobilised lower and 18 immobilised upper limbs. Limb immobilisation ranged from 12 h to 56 days. Isometric muscle strength and muscle size declined following immobilisation; however, change magnitude was greater for strength than size. Evoked resting twitch force decreased for lower but increased for upper limbs. Rate of force development either remained unchanged or slowed for lower and typically slowed for upper limbs. Twitch relaxation rate slowed for both lower and upper limbs. Central motor drive typically decreased for both locations, while electromyography amplitude during maximum voluntary contractions decreased for the lower and presented mixed findings for the upper limbs. Trends imply faster rates of NMF loss relative to size earlier in immobilisation periods for all outcomes.

**Conclusions:**

Limb immobilisation results in non-uniform loss of isometric muscle strength, size and NMF over time. Different outcomes between upper and lower limbs could be attributed to higher degrees of central neural control of upper limb musculature. Future research should focus on muscle function losses and mechanisms following acute immobilisation.

**Registration:**

PROSPERO reference: CRD42016033692.

**Electronic supplementary material:**

The online version of this article (10.1007/s40279-019-01088-8) contains supplementary material, which is available to authorized users.

## Key Points

**Table:** 

Following periods of immobilisation, muscular strength, muscle size and neuromuscular function decrease.
Strength declined similarly irrespective of immobilisation location; however, there were differences in the change to neuromuscular function between the upper and lower limbs.
Fixed joint methods of immobilisation incur greater changes in strength and neuromuscular function than methods allowing free joint movements.

## Background

### Rationale

Single-limb or whole-body immobilisation can occur as a consequence of injury, illness, frailty or surgery [1–3], in highly specific circumstances such as spaceflight [4] or merely due to reduced physical activity [5]. Such periods of immobilisation can be of different duration and occur at multiple time points across the lifespan. Regardless of the reason for immobilisation, it results in a decrease in muscle function and muscle volume resultant from mechanical unloading of the immobilised musculature, and as a consequence results in impaired capacity for activities of daily living and quality of life. The immobilisation studies reviewed within this paper therefore provide important insights into the functional, biochemical and physiological consequences of periods of inactivity that are commonly experienced after musculoskeletal injuries and during illness, especially when hospitalisation occurs. The improved understanding of the mechanisms and processes that contribute to the deterioration in function observed can then be used to develop evidence-based strategies to counteract these detrimental effects.

Significant muscle atrophy, evidenced by a decrease in muscle size at the whole muscle or single fibre level [6–8], occurs in response to immobilisation. Concomitantly, a reduction in muscle function is shown, most commonly quantified by a decrease in strength or the ability to volitionally produce force [9]. The loss in muscle strength during immobilisation is typically greater and occurs faster compared to the loss of muscle volume [9]. As such, muscle atrophy cannot fully explain the immobilisation-induced loss in muscle strength. Whilst muscle fibre cross-sectional area is a key factor in determining maximal force-generating capacity, muscle function and strength are also strongly influenced by neural mechanisms [10]. Therefore, it is plausible that changes in neural processes or neuromuscular function (NMF) may be responsible for the disproportionately higher loss in muscle strength relative to the reduction in muscle size (muscle mass or muscle volume) with immobilisation. Neuromuscular function is dependent on both peripheral and central processes, from the generation and transmission of neural activation signals within the central nervous system to the transmission to and action of the contractile apparatus. Therefore, changes in muscle excitability and contractility, as well as in central neural drive, may be important factors underlying the deterioration of muscle function and strength following limb immobilisation. Improved understanding of the magnitude and rate of immobilisation-induced changes in strength, muscle size and NMF may inform treatment and rehabilitation strategies for injured athletes as well as clinical, ageing and inactive populations.

### Objectives

The primary aim of this study was to systematically review the literature and quantify changes in isometric muscular strength, muscle size and NMF (e.g. muscle excitability and contractility, and central motor drive) following periods of enforced limb immobilisation in healthy adults. Secondary aims were to quantify the effect of: (1) the duration of immobilisation (short vs. long); (2) the method of immobilisation (fixed joint vs. freely moving joint); and (3) the location of immobilisation (lower vs. upper limb) on the induced muscle morphological, physiological and functional changes.

## Methods

### Protocol

The systematic review was undertaken in accordance with a predefined protocol (PROSPERO reference: CRD42016033692) and is reported in accordance with PRISMA reporting guidelines [11].

### Study Identification

A systematic literature search was performed in Medline, EMBASE, CINAHL, HMIC, SPORTDiscus and Web of Science. Forward (using Web of Science) and backward supplementary searching was also performed on all included studies. All citations from the literature searching were collated and de-duplicated in EndNote (Thomson Reuters V8).

Searches were conducted to include all studies published from the date of database inception to 13 December 2018. Terms for ‘human population’ were not included in the search strategy to limit the number of studies inadvertently missed due to title and abstract nomenclature. The search strategy took the following form:

(terms for immobilisation) AND (terms for methods of immobilisation) AND (terms for neuromuscular outcomes)

The full search strategy is provided in Electronic Supplementary Material Appendix S1.

### Study Selection

Two reviewers (MC and JVC) independently screened titles and abstracts of the retrieved citations according to predefined inclusion criteria (see Sect. 2.4). The inclusion criteria were piloted against 10% of the retrieved citations, and following agreement the remainder of the titles and abstracts were screened in duplicate. Full texts of included titles/abstracts were obtained and screened. A third author (JB) reviewed full-text articles when consensus on suitability was not met.

### Inclusion Criteria

Studies were included if measures of NMF and isometric strength made before and after a period of enforced immobilisation were reported in healthy adult (18 + years) humans. Included studies were not limited to randomised controlled trials as a large portion of the available literature used convenience sampling. Systematic reviews that met the inclusion criteria were also retained and their reference lists screened for studies meeting the inclusion criteria.

### Exclusion Criteria

Studies were excluded if the experiments used animal models or the human population was described as injured or not healthy to avoid extraneous influence of illness upon the immobilisation effects. Studies that used bed rest or whole-body immobilisation as their method of immobilisation were initially included due to the comparable loss of muscle size as presented by Dirks and colleagues [12]. However, these studies were later removed following a protocol amendment due to the potential interference of systemic changes and resultant effects on NMF. Studies were also excluded if the immobilisation was interrupted by any means such as removing the brace to test strength mid-way through the immobilisation period. If, however, these mid-point data were reported then the study was included with these mid-point data extracted and the duration of immobilisation was adjusted accordingly. Studies were also excluded if there was no measure of isometric strength since we used this outcome to evaluate the effectiveness of the immobilisation protocol used. A summary of the inclusion and exclusion criteria is presented in Table 1.

**Table 1:** Summary of inclusion and exclusion criteria

	Inclusion	Exclusion
Population	Healthy adult humans	Animal models or human populations described as injured or non-healthy
Intervention	Immobilisation by any means, e.g. brace, cast, ULLS, sling or any isolated body part	Bed rest or whole-body immobilisation, interference with immobilisation, e.g. interruptions
Comparator	n/a	
Outcomes	NMF, isometric strength	
Study design	Pre and post measures of NMF and isometric muscle strength following a period of enforced immobilisation	

*n/a* not applicable, *NMF* neuromuscular function, *ULLS* unilateral lower limb suspension

### Data Extraction

Data from studies meeting the inclusion criteria were extracted by one reviewer (MC) and checked by a second reviewer (JVC). Data pertaining to the main outcome measures, namely NMF, isometric strength and, if available, muscle size from before and after immobilisation were extracted using a standardised data extraction form. Only data pertaining to the immobilised limb were extracted; no data for the contralateral limb were extracted. Participant anthropometric and demographic characteristics, information on the method(s) of immobilisation and data collection procedures were also extracted. When numerical data were not reported in the text but reported in figures, extraction was conducted using InkScape 0.91 and GIMP2.0 using vector graphic principles.

Where multiple publications were identified that presented data from the same study (i.e. same group of participants and same intervention), the publication with the most relevant data was used as the main reference, with additional details extracted from the other publications as necessary.

### Assessment of Methodological Quality

Quality of the included studies was assessed by two authors (MC and JVC) and in the case of disagreement was resolved by a third author (JB). The methodological quality assessment was based on the Effective Public Health Practice Project (EPHPP) quality assessment tool [13] and adapted for use in this review. The subsections relating to confounders, intervention integrity and analysis (sections C, G, H in the EPHPP) were removed as not relevant to this research question. The evaluation of study design and selection bias was adapted for relevance to this research question. Each section was scored as weak (= 1), moderate (= 2) or strong (= 3). Overall study mark was calculated by summation of the section scores and used to categorise its methodological quality as being weak (= 4–6), moderate (= 7–9) or strong (= 10–12).

### Statistical Analysis and Data Synthesis

The studies were narratively synthesised. Data were ordered by the three main outcome measures (isometric muscle strength, muscle size and NMF) and sub-sectioned by location and method of immobilisation.

Published raw data were used to calculate the percentage change in the outcome measures from pre- to post-immobilisation ({post score − pre score)/ (pre score} × 100%) unless percentage changes were stated in the paper and therefore included as stated. The daily rate of change in isometric muscle strength, muscle size and NMF was calculated as the ratio between the percentage change and the number of days of immobilisation to generate comparative data across studies.

Pearson’s correlation coefficient was calculated to evaluate the strength of the relationships between changes in isometric muscle strength and the other extracted variables of interest. Scatterplots and tables of all raw data extracted from the included studies are provided in Electronic Supplementary Material Appendix S1–S9 and Tables S2–S10. Data are presented as ranges with medians unless otherwise stated.

## Results

### Search Results

**Fig. 1:**
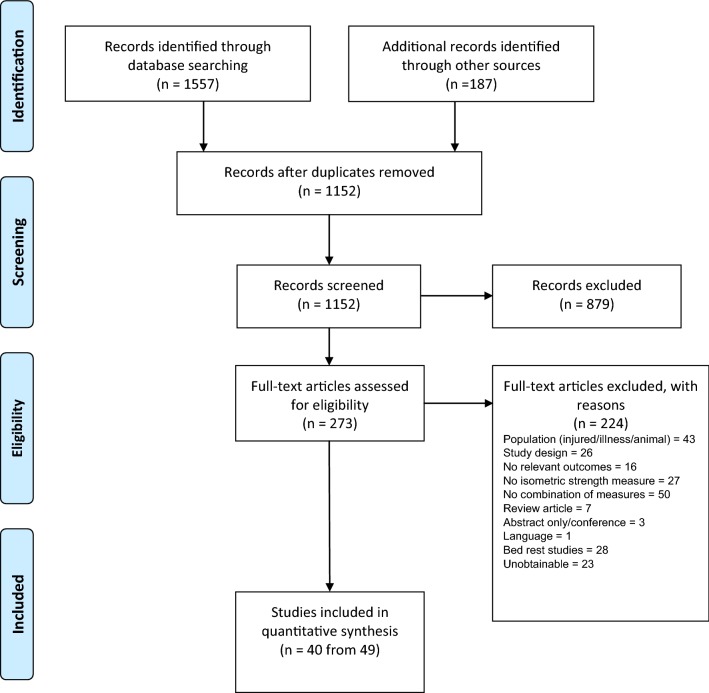
PRISMA flow diagram

In total 1744 studies were identified via the database and supplementary searches. After the removal of duplicates, 1152 unique references were entered for title and abstract screening. Of them, 273 studies underwent full text screening for eligibility. A total of 40 unique studies (49 citations [14–62]) met the inclusion criteria and were included in the final review (Fig. 1).

### Study Characteristics

A total of 431 participants were involved across the 40 included studies, and comprised 71% males (*n *= 308), 24% females (*n *= 102) and 5% sex not reported (*n *= 21). Across the studies, age ranged between 18.8 and 68.5 years (median 23 years). Four studies specifically recruited older participant groups for comparison with younger groups [25, 35, 38, 59]. The duration of immobilisation ranged from 0.5 to 35 days. In 93% of the studies, the duration of immobilisation was ≥ 7 days. A portion of the lower limb was partially immobilised in 22 studies and a portion of an upper limb was immobilised in 18 studies.

**Fig. 2:**
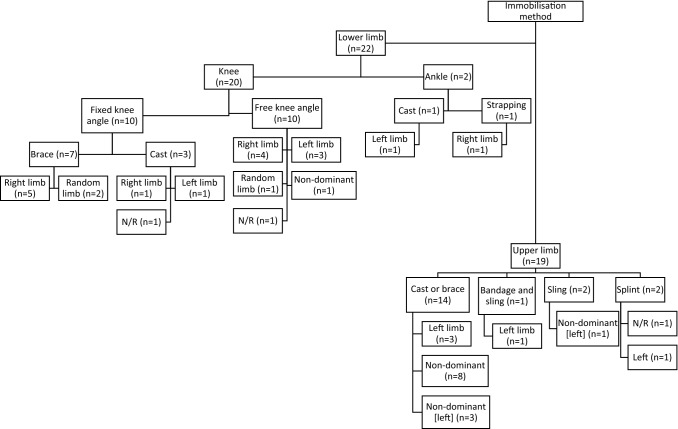
Summary of immobilisation methods and body segments.* N/R* not reported

Across the 40 studies, the following locations were immobilised: knee, ankle, elbow, wrist and finger. Immobilisation was achieved using cast, brace, sling, unilateral limb suspension (ULLS), strapping or splint. Some studies randomised the immobilised side (*n *= 4) whilst some specifically used non-dominant (*n *= 16) or predetermined to right (*n *= 11) or left (*n *= 8); one study did not report what side of the body was immobilised. A summary of the characteristics of all included studies is presented in Table 2. A dissection of immobilisation locations and methods used across the included studies is shown in Fig. 2.

**Table 2:** Summary of the characteristics of the included studies

Location	Immobilisation method	Study	Group no. (total no.)	Male/ female (young/old)	Age in years (SD) or [range]	Height in centimetres (SD)	Weight in kilograms (SD)	Body part (left/right)	Duration of immobilisation in days (total days in study if interrupted)
Lower limb	Brace	Hvid et al. [63] (Hvid et al. [37], Suetta et al. [55])	11	11 M (O)	67.2 (1.0)	178.8 (1.7)	87.7 (3.0)	Knee^a^	4
			11	11 M (Y)	24.3 (0.9)	180.4 (2.7)	74.3 (2.4)		
		Deschenes et al. [25]	10	10 M (O)	68.5 (1.6)	176.7 (1.3)	88.0 (2.2)	Leg(R)	7
			10	10 M (Y)	21.7 (1.1)	175.8 (2.8)	74.4 (4.2)		
		Deschenes et al. [27]	20	10 M	21.4 (0.8)	175.8 (2.8)	74.4 (4.2)	Leg(R)	7
				10 F	20.9 (0.2)	168.7 (1.3)	65 (3.6)		
		Deschenes et al. [26]	10	10 M	20.9 (1.3)	175.9 (5.4)	80.5 (19.2)	Leg(R)	7
		Deschenes et al. [28]	24	12 M	20.7 (0.3)	176.5 (2.0)	72.4 (2.5)	Leg(R)	7
				12 F	20.3 (0.3)	167.1 (2.3)	62.9 (1.3)		
		Davies et al. [21]	11	11 F	19.4 (0.9)	165.6 (6.4)	54.9 (5.1)	Leg(R)	7 (21)
		White et al. [61]	4	4 M	25 (7)	NR	NR	Leg(L)^b^	7 (14)
		Deschenes et al. [24]	10	6 M/4F	21 (0.4)	174 (2.3)	78.7 (7.3)	Leg(R)	14
		Hvid et al. [35] (Suetta et al. [57], Suetta et al. [56], Hvid et al. [36])	9	9 M (O)	67.3 (1.3)	178.7 (2.6)	84.8 (3.4)	Leg^a^	14
			11	11 M (Y)	24.4 (0.5)	181.4 (1.8)	72.2 (2.3)		
		Oates et al. [45]	5	2 M/3F	23.9 (2.2)	176 (6)	73 (8)	Knee^a^	14
	ULLS	Berg and Tesch [14]	10	10 M	24 (3)	186 (7)	75.0 (5.0)	Leg^a^	10
		de Boer et al. [22] (de Boer et al. [23])	9 (17)	9 M	19.1 (0.6)	179.3 (4.7)	72.4 (8.6)	Leg^b^	14 (23)
		Seynnes et al. [53] (Seynnes et al. [54])	8 (16)	8 M	19 (0.2)	179 (2)	70.3 (2.1)	Leg(R)	14 (23)
		Hotta et al. [34]	5 (11)	5 M	21.6 (3.4) _n=11_	170.2 (5.7) _n=11_	60.8 (9.4) _n=11_	Leg	20
		Campbell et al. [15]	8 (16)	8 M	23 (2.2)	NR	NR	Leg(R)	21
		Horstman et al. [33]	6	6 M	21 (1)	187 (6)	79.0 (9.0)	Leg(R)	21
		Schulze et al. [48]	8 (32)	8 M	27.1 (3)	181 (2)	77.3 (5.3)	Leg(L)	21
		Seynnes et al. [52]	6	6 M	23 (2)	187 (7)	79 (9)	Leg(R)	24
		Cook et al. [19] (Cook et al. [20])	8 (16)	4 M/4F	18.8 (1.0)	168.3 (12.2)	63.9 (14.2)	Leg(L)	30
		Tesch et al. 2004 [58]	11 (21)	7 M/4F	40 (9)	176 (9)	80 (14)	Leg(L)	~35
Ankle	Brace/cast	Lundbye-Jensen and Nielsen [42]	12	9 M/3F	25 (6)	NR	NR	Foot(L)	14
		Gondin et al. [32]	8 (17)	8 M	25.8 (1.6)	176.4 (2.0)	70.0 (2.6)	Foot(R)	~14
Upper limb	Brace/cast	Inada et al. [39]	10 (30)	10 M	29.5 (4.2) _n=30_	171.1 (4.4) _n=30_	66.5 (6.8) _n=30_	Hand(L)	0.5
		Ngomo et al. [44]	11	NR	26.5 (4.3)	NR	NR	Wrist and fingers^b^	4
		Clark et al. [16]	10 (19)	5 M/5F	21.9 (0.5)	169.4 (3.2)	77.7 (5.0)	Forearm^b^	7 (21)
		Fuglevand et al. [31]	11	8 M/3F	(22–38)	NR	NR	Hand(L)^b^	7 (21)
		Lundbye-Jensen and Nielsen [41]	10	6 M/4F	24 (6)	NR	NR	Forearm(L)^b^	7
		Seki et al. [49]	5	5 M	(22–29)	NR	NR	Hand(L)	7
		Karolczak et al. [40]	7(18)	7 M	30.43 (7.66)	179.50 (6.24)	78.92 (3.54)	Upper limb^b^	14
		Urso et al. [59]	28	20 M (O)	67 (4)	175.9 (1.8)	88.3 (3.8)	Hand^b^	14
				8 M (Y)	21 (2)	177.8 (2.5)	81.9 (5.5)		
		Vaughan [60]	6	4 M/2F	31.2 (25–37)	NR	NR	Upper limb^b^	14
		Clark et al. [18]	11 (20)	6 M/5F	20.5 (0.4)	173.9 (3.5)	69.9 (4.3)	Forearm^b^	21
		Farthing et al. [29]	10 (30)	2 M/8F	22.2 (2.8)	169.7 (8.8)	72.5 (24.4)	Forearm(L)^b^	21
		Farthing et al. [30]	7 (14)	1 M/6F	22.7 (4.4)	162.5 (9.3)	65.8 (13)	Forearm(L)^b^	21
		Seki et al. [50] (Seki et al. [51])	7 (9)	7 M	(21, 22)	NR	NR	Hand(L)^b^	21 (42)
		Clark et al. [17]	15 (44)	8 M/7F	21.2 (3.5)	170.8 (10.9)	70.1 (10.8)	Forearm^b^	28
		Yue et al. [62]	10	NR	(19–27)	NR	NR	Arm(L)	28
		Sale et al. [47]	11	11 M	(19–22)	NR	NR	Arm^b^	35
	Sling	Pearce et al. [46]	9 (28)	4 M/5F	25.3 (8.7)	173.6 (9.1)	62.5 (10.1)	Arm(L)^b^	21
		Magnus et al. [43]	8 (25)	2 M/6F	20.3 (1.8)	170.6 (10.3)	83.2 (28.4)	Arm(L)^b^	27.8 ± 2.3

*F* female, *L* left, *LB* leg brace/cast, *LU* leg ULLS, *M* male, *NR* not reported, *O* old people, *R* right, *UL* upper limb, *ULLS* unilateral limb suspension, *Y* young people, *~* approximately stated or mean value given

^a^Randomised limb

^b^Non-dominant limb

### Methodological Characteristics

#### Neuromuscular Function

**Fig. 3:**
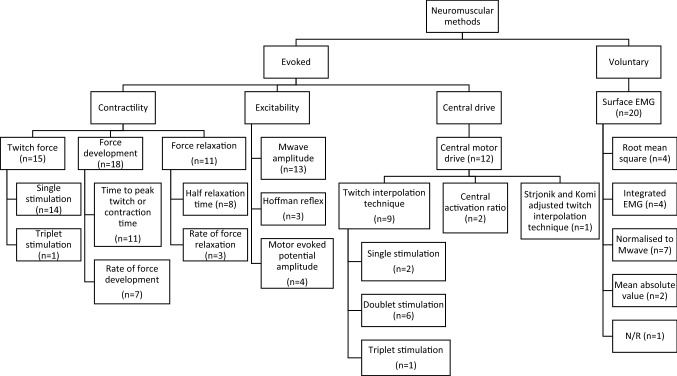
Summary of methods used in the studies to evaluate neuromuscular function.* EMG* electromyography,* N/R* not reported

A summary of the methods and measures used to assess NMF is presented in Fig. 3. A more in-depth explanation can be found in Electronic Supplementary Material Table S11.

#### Muscle Strength

All included studies measured isometric muscle strength as per the inclusion criteria. Isometric muscle strength during maximal voluntary contractions (MVC) was measured using: (1) a commercially available dynamometer (23 studies); (2) hydraulic recording systems (two studies); (3) load cells (one study); (4) strain gauges (eight studies); and (5) force transducers (six studies). One study did not report the method used to evaluate muscle strength. When quantifying muscle strength, 20 studies reported the ‘peak’ or ‘max’, ‘highest’, ‘greatest’, ‘best’, or ‘largest’ force value, three studies reported the ‘plateau’ force level, and the remaining studies (*n *=26) did not state how muscle strength was quantified.

#### Muscle Size

A total of 22 studies measured changes in muscle size from before to after immobilisation. Three studies quantified alterations in muscle fibre cross sectional area, two studies applied an anthropometric model using skinfolds combined with limb circumference measures, four studies used an ultrasound measurement of muscle thickness, one used dual-energy x-ray absorptiometry (DXA) to measure lean muscle mass, one used X-ray computerised axial tomography for whole muscle cross-sectional area, and 11 used magnetic resonance imaging (MRI). The MRI studies used different combinations of MR field strength, slice thickness and slice-to-slice intervals (see Electronic Supplementary Material Table S1).

### Methodological Quality

Full results from the methodological assessment can be found in Table 3. Overall, the methodological quality of the studies included was evaluated as ‘moderate’. No included study was rated as ‘strong’, while four studies were classified as ‘weak’ according to our methodological quality assessment. Common sources of weakness were: (1) poor reporting of participant inclusion criteria (*n *=22); (2) no randomisation of the immobilised limb (*n *= 36); and (3) the participant (*n *=40) or outcome assessors (*n *=40) were not blinded to the research question.

**Table 3:** Methodological quality assessment

Location	Immobilisation method	Study	Selection bias	Study design	Blinding	Withdrawals/ dropouts	Overall rating
Lower limb	Brace	Hvid et al. [63] (Hvid et al. [37], Suetta et al. [55])					
		Deschenes et al. [25]					
		Deschenes et al. [27]					
		Deschenes et al. [26]					
		Deschenes et al. [28]					
		Davies et al. [21]					
		White et al. [61]					
		Deschenes et al. [24]					
		Hvid et al. [35] (Suetta et al. [57], Suetta et al. [56], Hvid et al. [36])					
		Oates et al. [45]					
	ULLS	Berg & Tesch [14]					
		de Boer et al. [22] (de Boer et al. [23])					
		Seynnes et al. [53], (Seynnes et al. [54])					
		Hotta et al. [34]					
		Campbell et al. [15]					
		Horstman et al. [33]					
		Schulze et al. [48]					
		Seynnes et al. [52]					
		Cook et al. [19] (Cook et al. [20])					
		Tesch et al. [58]					
Ankle	Brace/cast	Lundbye-Jensen & Nielsen [42]					
		Gondin et al. [32]					
Upper limb	Brace/cast	Inada et al. [39]					
		Ngomo et al. [44]					
		Clark et al. [16]					
		Fuglevand et al. [31]					
		Lundbye-Jensen & Nielsen [41]					
		Seki et al. [49]					
		Karolczak et al. [40]					
		Urso et al. [59]					
		Vaughan [60]					
		Clark et al. [18]					
		Farthing et al. [29]					
		Farthing et al. [30]					
		Seki et al. [50], (Seki et al. [51])					
		Clark et al. [17]					
		Yue et al. [62]					
		Sale et al. [47]					
	Sling	Pearce et al. [46]					
		Magnus et al. [43]					

*ULLS* unilateral limb suspension

= weak,  = moderate,  = strong

### Synthesis

**Fig. 4:**
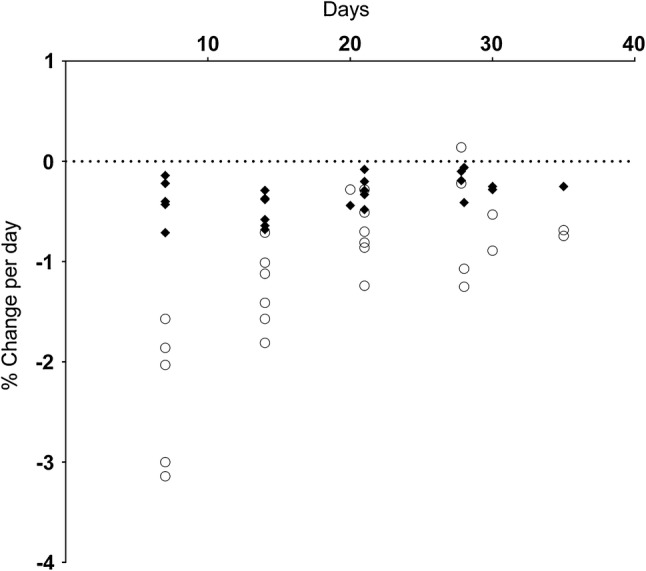
Muscle strength and muscle size change per day. Muscle strength changes are shown as open circles, muscle size changes as closed diamonds

**Fig. 5:**
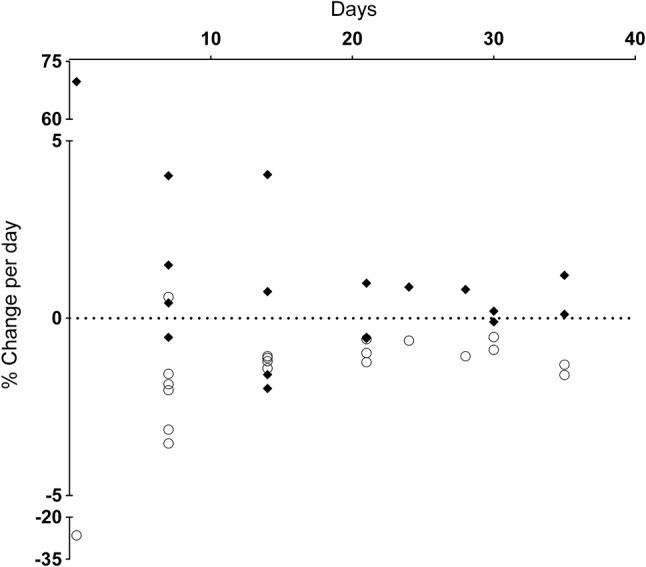
Muscle strength and resting twitch force change per day. Muscle strength changes are shown as open circles, resting twitch force changes as closed diamonds

**Fig. 6:**
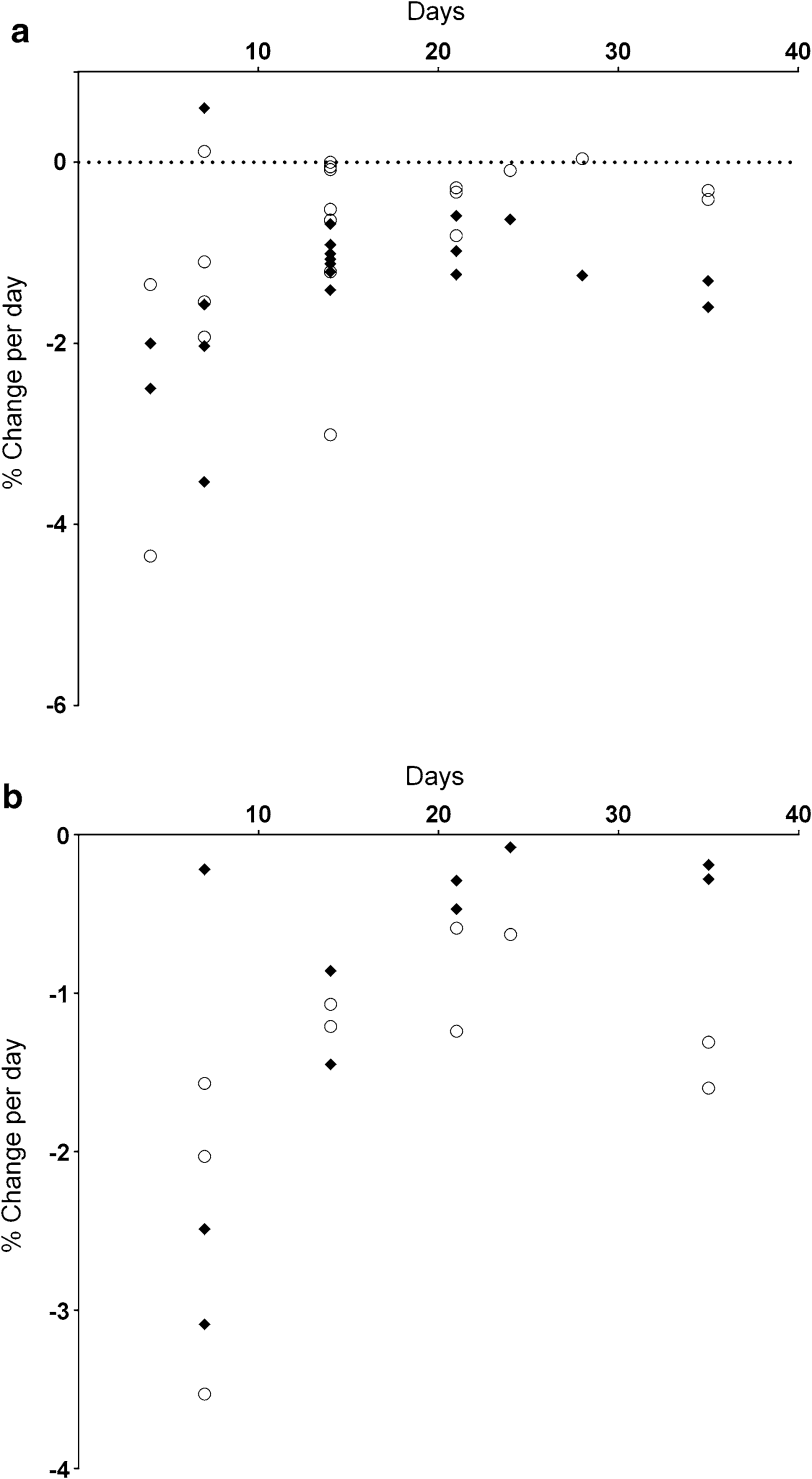
Muscle strength and rate of force development change per day (**a**) and muscle strength and rate of force relaxation change per day (**b**). Muscle strength changes are shown as open circles, force development or relaxation changes as closed diamonds

**Fig. 7:**
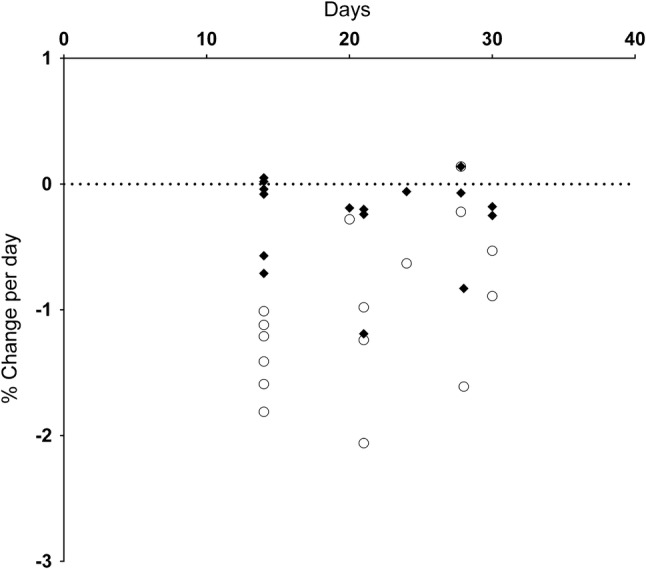
Muscle strength and central drive change per day. Muscle strength changes are shown as open circles, central drive changes as closed diamonds

**Fig. 8:**
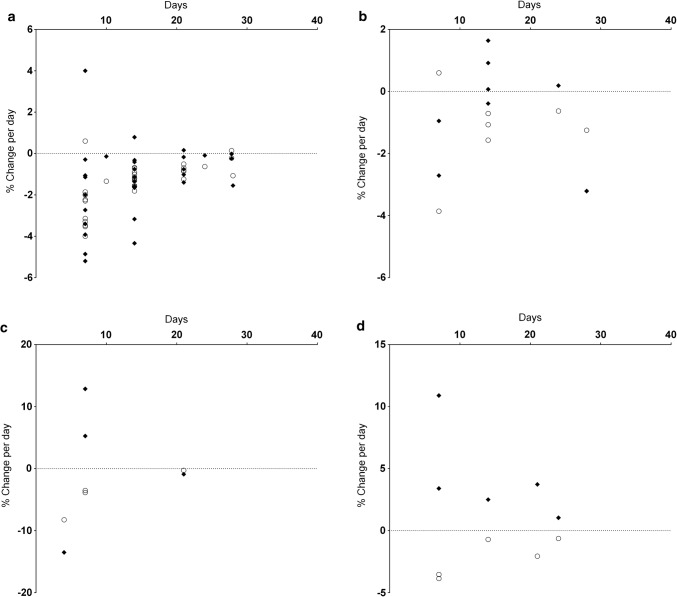
Muscle strength and EMG change per day (**a**), muscle strength and Mwave amplitude change per day (**b**), muscle strength and motor evoked potential change per day (**c**), muscle strength and maximal Hoffman reflex amplitude change per day (**d**). Muscle strength changes are shown as open circles, other variables as closed diamonds. *EMG* electromyography

All outcome measure data are reported separately by limb, immobilisation method and, where possible, muscle action. The relationship between isometric muscle strength changes and the remaining variables of interest are presented in the accompanying scatterplots (Figs. 4, 5, 6, 7, and 8), in which only data from those studies with both variables are displayed.

#### Muscle Strength

##### Lower Limb

Knee extensor strength was reduced post-immobilisation using a brace (*n *= 14: range − 1.1 to − 4.0%·day^−1^; median − 2.0%·day^−1^) and ULLS (*n *= 7: range − 0.5 to − 1.3%·day^−1^; median − 1.0%·day^−1^).

Plantar flexor strength declined following the use of casts (*n *= 3: range − 1.6 to − 2.0%·day^−1^; median − 1.8%·day^−1^) and using ULLS (*n *= 6: range − 0.3 to − 0.9%·day^−1^; median − 0.7%·day^−1^). In the studies that specifically cast the ankle, both observed plantar flexor strength declined (*n *= 2: − 1.1%·day^−1^ and − 1.2%·day^−1^). Dorsiflexor strength was only measured in one study, which showed an overall decline (− 1.6%·day^−1^).

##### Upper Limb

Upper limb immobilisation caused a loss in strength of the elbow flexors (*n *= 3: − 0.9 to − 1.3%·day^−1^; median − 1.2%·day^−1^). By contrast, the loss of elbow flexor strength when immobilisation was achieved using a sling was variable across studies (*n *= 2: + 0.1%·day^−1^ increase and − 0.3%·day^−1^ decrease). Elbow extensor strength declined across all studies using both brace (*n *= 3, − 0.6 to − 1.3%·day^−1^; median − 1.1%·day^−1^) and sling (*n *= 1, − 0.2%·day^−1^) immobilisation methods.

Wrist flexor strength decreased across all studies (*n = *6: range − 0.5 to − 3.9%·day^−1^; median − 1.8%·day^−1^), while a single study measured a decrease in wrist extensor strength (− 3.5%·day^−1^) following use of casts.

Immobilisation of the finger and thumb muscles via brace or cast resulted in both increases and decreases (*n = *11: range + 0.6%·day^−1^ increase to − 26.5%·day^−1^ decrease; median − 1.6%·day^−1^).

#### Muscle Size

##### Lower Limb

Studies using a fixed-angle brace model observed a decline in muscle size in the muscles above the knee (*n =* 5: range − 0.2 to − 0.6%·day^−1^; median − 0.4%·day^−1^) and below the knee (*n = *4: range − 0.4 to − 0.7·day^−1^; median − 0.6%·day^−1^).

Following lower limb suspension, muscle size decreased above the knee (*n = *5: range − 0.3 to − 0.5%·day^−1^; median − 0.3%·day^−1^) and below the knee (*n = *6: range − 0.3 to − 0.4%·day^−1^; median − 0.4%·day^−1^).

##### Upper Limb

Declines in upper limb muscle size were established after brace (*n =* 9: range − 0.1 to − 0.7%·day^−1^; median − 0.2%·day^−1^) and sling (*n =* 3: range − 0.1 to − 0.3%·day^−1^; median − 0.2%·day^−1^) immobilisation.

The rate of strength loss was greater than the rate of muscle size loss across all studies where both parameters were available (Fig. 4).

#### Neuromuscular Function

##### Muscle Contractility

*Resting Twitch Force*: *Lower limb* Knee extensor twitch force (Fig. 5) decreased following bracing (*n = *2: − 1.6 and − 2.0%·day^−1^) but the rate of change both increased and decreased following ULLS (*n = *3: range + 0.2%·day^−1^ increase to − 0.6%·day^−1^ decrease; median − 0.5%·day^−1^).

Plantar flexor twitch force increased following knee (*n = *2: + 0.4 and + 1.5%·day^−1^) and ankle (*n = *2: + 0.8 and + 4.1%·day^−1^) bracing and exhibited both an increase and a decrease following ULLS (*n =* 2, + 0.1%·day^−1^ increase and − 0.1%·day^−1^ decrease).

*Upper Limb* The amplitude of resting twitch force evoked in wrist flexor muscles declined (*n = *2: − 0.4 and − 0.5%·day^−1^) but increased in the hand musculature (*n = *5: range + 0.1%·day^−1^ to + 69.8%·day^−1^; median + 1.2%·day^−1^). Elbow flexor twitch force increased in one study (+ 0.81%·day^−1^). All upper limb measures utilised brace or cast immobilisation (Fig. 5).

*Force Development and Relaxation*: Measures of resting twitch force development and relaxation were reported either as duration or as a rate of change. For the purposes of data summary, all duration data were inverted so that an increase in duration, indicating an impaired response, was expressed as a negative, and therefore a decrease in % change per day indicates an ‘impaired’ response.

*Force Development*: *Lower limb* Knee extensor force development time (Fig. 6a) either remained unchanged or slowed down following bracing (*n = *4: range 0 to − 4.4%·day^−1^, median − 0.7%·day^−1^) and ULLS (*n =* 3: range − 0.3 to − 3.0%·day^−1^, median − 0.8%·day^−1^). The time for plantar flexor force development was also slower following knee bracing (*n = 2*: − 1.5 and − 1.9%·day^−1^), ULLS (− 0.1%·day^−1^) and ankle brace (*n = 2,* − 0.1 and − 1.2%·day^−1^).

*Upper limb* Immobilisation resulted in slower resting twitch force development time (Fig. 6a) in the wrist flexors (*n = *2: − 0.1 and − 1.0%·day^−1^) and finger and thumb muscles (*n = *4: range − 0.3 to − 1.1%·day^−1^, median − 0.4%·day^−1^). One study measured a slowing of elbow extensor force development (− 0.5%·day^−1^) whilst elbow flexor force development displayed both increase and decrease (*n = *3: range + 0.04%·day^−1^ increase to − 0.6%·day^−1^ decrease, median − 0.4%·day^−1^).

*Force Relaxation*: *Lower limb* The studies reported a wide range of change across the lower limb (Fig. 6b), while one study showed an increase in knee extension relaxation time following ULLS (− 0.5%·day^−1^). Two studies showing an increase in plantar flexor relaxation time following brace immobilisation (*n = *2: − 0.8 and − 1.5%·day^−1^), while a single study observed a decrease following ULLS (+ 0.1%·day^−1^). Ankle immobilisation also slowed relaxation (*n = *2: − 0.9 and − 1.5%·day^−1^).

*Upper limb* Force relaxation (Fig. 6b) increased in the wrist flexors (− 0.2%·day^−1^), while finger and thumb relaxation was also prolonged (*n = *3: range − 0.2 to − 0.3%·day^−1^; median − 0.3%·day^−1^).

*Central Motor Drive*: *Lower limb* Central drive (Fig. 7) of the knee extensors decreased following bracing (*n = *2: − 0.1 and − 0.7%·day^−1^). Comparable decreases in the knee extensors were observed following ULLS, although one of five studies observed an increase (*n = *5: range + 0.1%·day^−1^ increase to − 0.2%·day^−1^ decrease; median − 0.2%·day^−1^). Similarly, the change following ULLS in the plantar flexors displayed both increased and decreased values (*n = *4: range + 0.02%·day^−1^ increase to − 0.3%·day^−1^ decrease; median − 0.1%·day^−1^). Following ankle immobilisation, central drive decreased (*n = *2: − 0.3 and − 0.6%·day^−1^).

*Upper limb* Central drive (Fig. 7) to the wrist flexors decreased following bracing (*n = *3: range − 0.8 to − 1.2%·day^−1^; median − 1.1%·day^−1^). Central drive to elbow flexors decreased (− 0.1%·day^−1^) but increased in elbow extensors (+ 0.1%·day^−1^) following a sling protocol.

*Volitional Surface EMG Activity*: *Lower limb* The amplitude of knee extensor EMG activity (Fig. 8a) during a maximal manoeuvre declined following bracing in all but one study (*n = *9: range + 0.8%·day^−1^ increase to − 5.2%·day^−1^ decrease; median − 1.1%·day^−1^) and ULLS altered EMG similarly with decreased activity (*n = *4: range − 0.1 to − 1.0%·day^−1^; median − 0.5%·day^−1^).

Plantar flexor EMG activity declined following knee bracing (− 0.4%·day^−1^), ULLS (*n = *3: range − 0.1 to 1.7%·day^−1^; median 1.4%·day^−1^) and ankle immobilisation (− 1.3%·day^−1^).

*Upper Limb* EMG activity (Fig. 8a) following bracing declined in the elbow flexors (*n = *3: range − 1.6 to − 3.2%·day^−1^; median − 1.6%·day^−1^), elbow extensors (*n = *2: − 0.8 and − 4.3%·day^−1^), wrist flexors (− 3.4%·day^−1^) and wrist extensors (− 2.7%·day^−1^). Sling immobilisation also induced a decrease in EMG activity of elbow flexors (− 0.6%·day^−1^) and elbow extensors (− 6.6%·day^−1^). EMG activity of finger and thumb muscles exhibited both increased and decreased findings (*n =* 3: range + 3.3%·day^−1^ increase to − 3.6%·day^−1^ decrease; median − 0.6%·day^−1^).

##### Muscle and Corticospinal Excitability

*Compound Muscle Action Potential*: *Lower limb* The amplitude of the compound muscle action potential (M_wave_) evoked post-immobilisation (Fig. 8b) exhibited an increase in the plantar flexors following ULLS (*n =* 3: range + 0.2 to + 1.3%·day^−1^; median + 0.6%·day^−1^) and both increases and decreases following ankle immobilisation (*n = *3: range + 0.2%·day^−1^ increase to − 0.4%·day^−1^ decrease; median − 0.3%·day^−1^).

*Upper limb* Across the seven studies measuring the M_wave_ evoked in upper limb muscles (Fig. 8b), there were amplitude decreases in both wrist flexors (− 1%·day^−1^) and elbow flexors (− 3.2%·day^−1^), with both increases and decreases in the finger and thumb muscles (*n =* 5: range + 1.6%·day^−1^ increase to − 2.7%·day^−1^ decrease; median + 0.1%·day^−1^). All studies utilised the brace/cast method.

*Motor Evoked Potential*: Changes in motor evoked potential (MEP) amplitudes were only measured in upper limb muscles (Fig. 8c). Elbow flexor MEP amplitude decreased following a sling protocol (− 0.1%·day^−1^) and finger muscles exhibited a decrease following casting (− 13.5%·day^−1^). MEP amplitudes registered in wrist flexors increased following brace/cast protocols (*n =* 2: + 5.3 and + 12.8%·day^−1^).

*Hoffmann Reflex*: *Lower limb* The amplitude of the maximal Hoffman reflex (Hmax) evoked in plantar flexors increased following ULLS (*n = *2: + 1.0 and + 2.5%·day^−1^; Fig. 8d).

*Upper limb* Hmax measured from wrist flexors increased after cast immobilisation (*n =* 3: range + 3.4 to + 10.9%·day^−1^; median + 3.7%·day^−1^; Fig. 8d).

#### Correlation

**Fig. 9:**
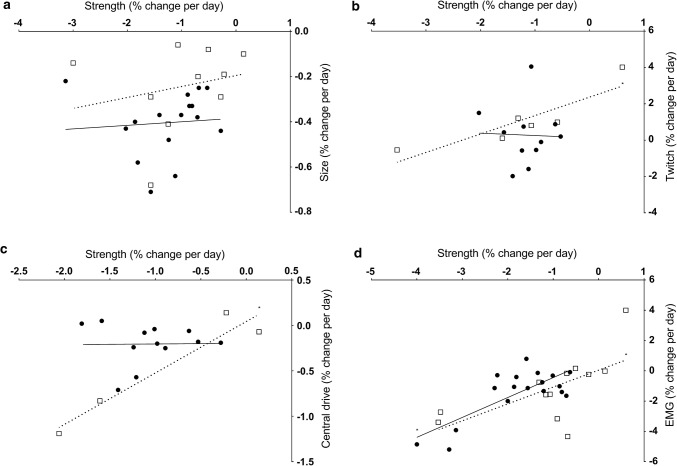
Correlation between muscle strength and size change per day (**a**), muscle strength and resting twitch force (**b**), muscle strength and central drive (**c**), muscle strength and EMG (**d**). Lower limb values are shown as circles with a solid line, upper limb values as squares with a dotted line. Significant correlations are indicated with an asterisk (*). *EMG* electromyography

There was no significant relationship between the rate of change in muscle strength and muscle size in response to either upper or lower limb immobilisation (Table 4, Fig. 9a). There was, however, a significantly positive relationship between the change in upper limb muscle strength and the change in voluntary activation of these muscles (*r* = 0.96, *p* = 0.04); no such relationship was found for the lower limb (Fig. 9b). Similarly, there was a positive and significant relationship between the rate of change in muscle strength and evoked twitch force with immobilisation for the upper (*r* = 0.88, *p* = 0.02) but not the lower limb (Fig. 9c). Finally, the rate of decline in muscle strength with immobilisation was significantly positively related to changes in EMG amplitude during maximal volitional isometric efforts in both the upper and lower limbs (upper *r* = 0.64, *p* = 0.03; lower *r* = 0.76, *p* < 0.001; Fig. 9d). Full graphical results from the correlation analysis can be found in Electronic Supplementary Material Figs. S1–S9.

**Table 4:** Relationship between muscle strength loss and other parameters in the upper and lower limbs

Experimental measure [% day^−1^]	Pearson’s correlation coefficient	
	Lower limb	Upper limb
Strength per day vs		
Size per day	0.08	0.23
Twitch force per day	− 0.03	0.88*
Force development per day	0.45	− 0.81*
Relaxation per day	0.80	− 0.57
Voluntary activation per day	0.01	0.96*
EMG per day	0.76*	0.64*
H_max_ per day	–	− 0.31
M_wave_ amplitude per day	0.72	− 0.36
MEP amplitude per day	–	0.53

*EMG* electromyography, *Hmax* Hoffman reflex, *MEP* motor evoked potential

*p < 0.05

#### Summary

**Fig. 10:**
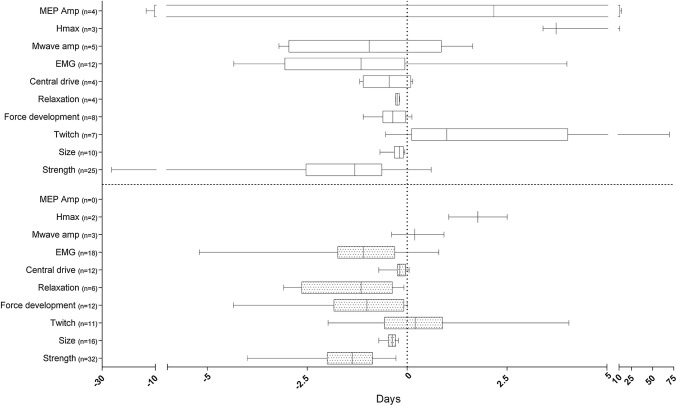
Box plot graph showing the minimum, first quartile, median, third quartile and maximum of the immobilisation-induced changes per day of the investigated measures for strength, muscle size and neuromuscular function presented individually for lower (bottom panel) and upper (top panel) limb. Values shown are median/range. *Amp* amplitude, *EMG* electromyography, *Hmax* Hoffman reflex, *MEP* motor-evoked potential, *n* number

A full overview of the changes per day for strength, muscle size and NMF split by location of immobilisation is presented in Fig. 10.

## Discussion

### Summary of Evidence

This is the first systematic review to consider the contribution of both muscle atrophy and deterioration in NMF to the loss of isometric muscle strength following immobilisation. The extracted data present strong evidence that the decrease in muscle size (i.e. muscle atrophy) cannot fully explain the functional loss, especially in the early phase of immobilisation. Periods of segmental human body immobilisation do result in decreased isometric muscular strength and size, but these changes occur alongside changes in both peripheral and central NMF, quantified by decreased muscle fibre excitability (Mwave amplitude) and contractility (decreased rate of force development and relaxation), decreased spinal (Hmax) and corticospinal excitability (MEP amplitude), and reduced central motor drive (increased resting twitch force amplitude, decreased voluntary activation) to the muscles. Changes in NMF appear to differ depending on immobilisation location, with upper limb immobilisation resulting in greater central changes and lower limb immobilisation in greater peripheral adaptations. While location of immobilisation appears to modulate the effects of immobilisation, the impact of joint action (extension vs. flexion) remains unclear due to a lack of evidence in the extensor muscles. Below, specific findings in relation to the aims of the systematic review are summarised and discussed individually.

#### Neuromuscular Factors Contribute to Decline in Muscle Strength

Muscle strength declined from before to after immobilisation in all but one study, while muscle size declined in all studies across both the lower and upper limbs. The weak, non-significant relationship between changes in muscle size and strength corroborate the notion that muscle atrophy contributes only partially to the functional loss. A strong positive correlation between the loss in muscle strength and decreases in central drive, increased resting twitch amplitude and decreased volitional EMG indicate greater influence of central NMF changes during upper limb than lower limb immobilisation.

In 22 of the 40 analysed studies, resting twitch force amplitude increased following periods of immobilisation. Interestingly, greater twitch force amplitude increases were observed in those studies where a greater reduction in central drive was also evident, suggesting maintenance of contractile function in the periphery alongside a clear attenuation in the central processes. A decrease in resting twitch amplitude was reported in the remaining 42% of studies, accompanied by lower rates of twitch force development and relaxation highlighting the detrimental effects of immobilisation on muscle contractility. Potential myofibrillar mechanisms underlying these functional changes may have included increases in intracellular calcium concentration [64], reductions in Ca^2+^-ATPase activity and Ca^2+^ uptake; decrease in protein synthesis rates [65], and increased dysfunction of myofibrillar and sarcoplasmic proteins [66]. Further investigation of the effect of immobilisation on calcium kinetics is warranted to improve understanding of the implicated cellular mechanisms.

The decline in contractile function must also be considered alongside the observation across the majority of studies that central motor drive was decreased following periods of immobilisation (− 0.2%·day^−1^ pooled median value). The current analysis pointed to differential effects of immobilisation on central neural drive modulation to muscles of the upper and lower limb; the pooled lower limb median value was 0.2%·day^−1^ loss of voluntary drive in comparison to 0.8%·day^−1^ loss in the upper limb. The decline in central drive was also observed in parallel with decreased volitional EMG amplitude during post-immobilisation maximal contractions. Central neural mechanisms appear to be a key component in the decline in NMF during and after limb immobilisation, especially in the upper limb. This conclusion is further corroborated by previous observations of no change or a decrease in resting membrane potential and no change in acetylcholinesterase activity in neuromuscular junctions after 4 weeks of immobilisation [65]. As highlighted in Sect. 3, there appears to be a wide variation in the effects of limb immobilisation on Mwave amplitude (an increase of + 1.64%·day^−1^ to a decrease of − 3.21%·day^−1^), which is indicative of peripheral muscle excitability, likely at least in part related to the different immobilisation locations and techniques employed in these studies. This makes it difficult to generate a clear conclusion or to speculate about possible underlying mechanisms. However, in line with the present analysis, recent evidence of neuromuscular plasticity during immobilisation [16] and of cross-education during retraining after immobilisation [67] point to decreased corticospinal drive as a primary mechanism in the reduction in muscular function and performance. Mechanisms implicated in the degenerative effects of short-term immobilisation include increased excitability of corticospinal networks (MEP and H-reflex amplitudes), intracortical inhibition (prolonged silent period) as well as interhemispheric interactions (motor irradiation).

A key finding of this review is that the greatest changes in all variables are occurring in the earliest stages of immobilisation, a finding similar to previous work investigating the effects of immobilisation on muscle protein synthesis [68]. When the relative changes in the measures of strength and NMF were plotted against the number of days of immobilisation, similar trends were found, with the greatest change occurring within the first week of immobilisation. It is important to note that this finding does not suggest that less immobilisation time elicits a greater change but that potentially the greatest rate of change is happening during the initial period of immobilisation, after which the rate of change plateaus. These data also suggest a greater contribution of NMF loss to declines in strength in the initial stages of immobilisation, whilst changes in muscle size dominate in the later stages. Analogously, it is well accepted that strength gains in the early stages of resistance training are predominantly related to neural factors as well as intracellular ionic changes (Ca^2+^ accumulation; [69]) rather than muscle hypertrophy. Further investigation of the mechanisms underlying the immobilisation-induced changes in muscle size, muscle strength and NMF is warranted. On the basis of this review and the identified magnitude and rate of change, short duration (< 7-day) immobilisation protocols can be used to investigate strategies for attenuating the loss of strength, muscle size and NMF during and following a period of immobilisation.

#### Differential Changes in Lower versus Upper Limbs

Several key findings can be extracted from the comparison of immobilisation-induced changes between upper and lower limbs. Firstly, strength declined in all but one study, and a comparable relative change of 1.3%·day^−1^ was found in both the lower and upper limbs. On the other hand, the rate of size loss in lower limb muscles was double that in the in the upper limbs with all methods combined (0.4%·day^−1^ vs. 0.2%·day^−1^) in parallel with greater deterioration in contractile function of the lower limb muscles (decline in rate of twitch force development and relaxation changes). In contrast, the decrease in voluntary activation and the increase in resting twitch force were higher following upper limb immobilisation. In summary, the similar declines in strength in upper and lower limb muscles were accompanied by greater reduction in central motor drive to the upper limb muscles, perhaps reflecting the greater degree of supraspinal control in the upper limbs [70]. On the other hand, the strength loss of lower limb muscles was accompanied by greater muscle atrophy and impaired contractility, suggesting stronger impact of immobilisation on peripheral mechanisms, potentially due to the previously observed [71] anti-gravity or postural muscles, i.e. the lower limb musculature with low frequency but long duration activation patterns appears to be more susceptible to unloading than the upper.

#### Effect of Immobilisation Method

Differential effects due to a variation in methods of immobilisation can be inferred from examination of the lower limb immobilisation studies assessing fixed angle versus free joint angle immobilisation techniques, e.g. brace and cast versus ULLS. Immobilisation involving joint fixation resulted in a greater strength loss. Muscle strength declines in both knee extensors and plantar flexors were almost twofold higher in studies using a fixed knee angle immobilisation method than those that used the ULLS method preserving a freely moving knee. This twofold difference in strength change was not, however, proportional to the differences in muscle size alterations (fixed model: − 0.4%·day^−1^ and − 0.6%·day^−1^ medians vs. free model: − 0.3%·day^−1^ and − 0.4%·day^−1^ median, upper and lower limb, respectively), which may be due to measuring the size loss across the whole group of muscles within the immobilised limb segment and disregarding the potential for differential effect size of immobilisation on muscles depending on fibre types [64] and muscle function. In a study using the ULLS method the biarticular rectus femoris muscle size loss was found to be ~ 50% less than that of the other monoarticular muscles of the thigh [15]. Previous work has also observed differential changes dependent on muscle length during immobilisation where muscles that are shortened degraded faster than when lengthened [66]. The choice of joint angle for immobilisation using the brace or cast method therefore appears likely to play a large role in outcomes.

The choice of method and location of immobilisation significantly impacts the magnitude of muscle function but not muscle size change. Multiple joint immobilisation is likely to produce the largest change in the NMF of segments consisting of both mono- and biarticular muscles. The changes in individual mono- and biarticular musculature within the immobilised muscle group should ideally be considered independently rather than pooled, due to the likelihood of differential change.

#### Effect of Participant Characteristics

Of the studies included, four compared outcomes in both old and young participants. For the NMF outcomes, the older participants had a greater percentage change between pre- to post-immobilisation compared to the younger participants, indicating a greater NMF decline. However, the data were equivocal with different magnitudes of strength and muscle size loss reported for older and young participants; larger [35], smaller [25, 63] and identical changes per day [59] in these parameters were observed between young and old participants.

Of the studies included, two [27, 28] recruited and compared outcomes in both males and females; a further 15 studies [16–19, 24, 29–31, 41–43, 45, 46, 58, 60] recruited both males and females but did not report their findings separately for sex. Typically, females lost more muscle strength, lost almost four times as much NMF (EMG), but lost less muscle size when compared to males.

Given the paucity of literature available on the differences between young and older participants and between the sexes, we would encourage future research in this area.

### Risk of Bias

Since some aspects of immobilisation studies cannot be blinded to the participant, inevitably all studies scored poorly on this aspect of the risk of bias assessment. However, the risk of bias could have been minimised more consistently throughout all the studies had the choice of limb immobilised been randomised and the outcome analysis blinded. This latter approach may have been used but was not reported explicitly by any of the included studies.

#### Data Heterogeneity

An important factor with potential to influence the size of reported changes is the choice of measurement technique for NMF, especially with regard to measures based on evoked responses such as twitch force and voluntary activation. Evoked resting twitch force was reported in 15 studies, but in these studies electrical stimuli were delivered to either nerve (*n *= 10) or muscle (*n *= 5) in single, doublet and triplet formats. Despite utilising the traditional twitch interpolation method for quantification of central motor drive/voluntary activation throughout the extracted literature, some studies utilised singlet rather than doublet stimuli for eliciting twitch responses during maximal contractions. The present analysis highlighted a lack of consensus for the best evaluation technique. This methodological heterogeneity prevented a meta-analysis of the included studies being performed.

The approach for measurement of muscle size also varied between studies and appears to be due mostly to techniques available to different research groups. Three different modalities were mainly employed – cross-sectional muscle fibre area, imaging techniques and anthropometric techniques. While this does not necessarily guarantee large disparities in the results, there were large differences in the application of each imaging technique. MRI was the most prevalent measurement technique within the included studies, but within this subsection (*n *= 11) different measurement parameters were used, such as slice thickness, number of slices and distance between slices. In some studies, these parameters were simply not reported, and many authors did not provide justifiable reasoning to clarify why choices were made. The lack of reporting could be considered a cause for concern as data can be easily manipulated to suit the outcome of choice by for example reducing the number of slices. Presentation of reliability data would have alleviated some aspects of risk of bias and would be encouraged for future research in this area. It was also not clear whether the method chosen to analyse the MRI data took account of intramuscular fat and connective tissue changes, which are expected to occur during immobilisation and if unaccounted for will lead to error in the estimation of muscle size.

Additionally, different parameters of the outcome measurements were extracted across the included studies for data presentation. For example, some citations presented the rate of twitch force development changes as absolute values while others presented only data normalised to body weight or as %MVC without the respective pre-normalised data. This approach can elevate the risk for potential bias. Therefore, to enhance the quality of future studies it is recommended to improve the transparency of methodological choices of measured parameters, grouping variables and normalisation procedures, in addition to reporting of absolute values and participant characteristics.

### Strengths and Weaknesses of the Review

This the first systematic review of the literature on immobilisation that analyses its effects on muscle atrophy, strength and function in parallel. There is a particular focus on the role of NMF and atrophy for the resultant loss in muscle strength, and variation across immobilised limb segments and immobilisation methods. All citations were independently screened by two reviewers.

Whilst the original search strategy captured most of the included citations, the remainder were found in forwards and backwards citation chasing. Studies found from supplementary searching were mostly those that used the term ‘unloading/unloaded’ or did not report the method of immobilisation within their title, abstract or keywords.

Studies that interrupted the immobilisation for taking measurements and those in which post-intervention measures were taken 24 h after the removal of the immobilisation method were excluded from the analysis. Where available, the earliest non-interrupted results were extracted and reported. This approach of excluding a number of studies completely or using only partial data from immobilisation interruptions was undertaken to minimise potential for skewing the presented findings.

Decisions regarding study or data inclusion and exclusion were, in some instances, extremely challenging, and it was not always possible to separate groups or participants within each study. Studies that involved control groups were often poorly reported, making it difficult to exclude their results from those of the intervention group. Future studies should explicitly report the methods, grouping variables (including clear participant characteristics for each sub group) and data manipulation procedures, and clearly state any previously published links between papers, particularly if the data reported are utilising the same participants, for example in the case of the group of studies represented by Hvid et al. [63].

A limitation, as with all systematic reviews, is publication bias or the selective publication of studies with positive findings. This may result in a distortion of the overall conclusions of any systematic review due to lack of access to data from non-published studies that typically report non-significant or dissentient findings.

## Conclusions and Implications

In conclusion, following periods of segmental limb immobilisation, isometric muscular strength, muscle size and NMF decrease. The magnitude of muscle strength loss is greater than muscle atrophy in the first few days of immobilisation, and loss of contractility (lower limb) and voluntary activation (upper limb) are important contributing factors especially in the early stages of immobilisation. Strength loss is similar between the upper and lower limbs, while size loss is twice as great in the lower limbs. Fixed joint methods of immobilisation are associated with greater changes in strength and NMF than methods allowing free joint movements. Only 10% of the included studies investigated the effects of immobilisation for less than 7 days, although the results indicate that this is the period in which the largest rate of change in all outcome measures occurs. Models using shorter durations would allow better understanding of the adaptations to immobilisation and of the role that different mechanisms, in particular those underlying NMF, play in the rapid decline in muscle strength during immobilisation.

### Data Availability Statement

Data and materials are available on request from the corresponding author.

## Electronic supplementary material

Below is the link to the electronic supplementary material.

Supplementary material 1 (DOCX 113 kb)

**Figure S1**. Correlation between muscle strength and size change per day. Lower limb values in circles with solid line, upper limb in squares with dotted line. Significant correlations indicated with an asterisk (*) (TIFF 137 kb)

**Figure S2.** Correlation between muscle strength and resting twitch force change per day. Lower limb values in circles with solid line, upper limb in squares with dotted line. Significant correlations indicated with an asterisk (*) (TIFF 126 kb)

**Figure S3.** Correlation between muscle strength and central drive change per day. Lower limb values in circles with solid line, upper limb in squares with dotted line. Significant correlations indicated with an asterisk (*) (TIFF 142 kb)

**Figure S4.** Correlation between muscle strength and EMG change per day. Lower limb values in circles with solid line, upper limb in squares with dotted line. Significant correlations indicated with an asterisk (*) (TIFF 137 kb)

**Figure S5.** Correlation between muscle strength and force development change per day. Lower limb values in circles with solid line, upper limb in squares with dotted line. Significant correlations indicated with an asterisk (*) (TIFF 134 kb)

**Figure S6.** Correlation between muscle strength and force relaxation change per day. Lower limb values in circles with solid line, upper limb in squares with dotted line. Significant correlations indicated with an asterisk (*) (TIFF 130 kb)

**Figure S7.** Correlation between muscle strength and Hmax change per day. Lower limb values in circles with solid line, upper limb in squares with dotted line. Significant correlations indicated with an asterisk (*) (TIFF 117 kb)

**Figure S8.** Correlation between muscle strength and Mwave amplitude change per day. Lower limb values in circles with solid line, upper limb in squares with dotted line. Significant correlations indicated with an asterisk (*) (TIFF 126 kb)

**Figure S9.** Correlation between muscle strength and motor evoked potential amplitude change per day. Lower limb values in circles with solid line, upper limb in squares with dotted line. Significant correlations indicated with an asterisk (*) (TIFF 129 kb)
