# Supplementary material for: Effect of Immobilisation on Neuromuscular Function In Vivo in Humans: A Systematic Review
Source: Sports Med. 2019 Mar 21;49(6):931–50. doi: 10.1007/s40279-019-01088-8 (PMC6548068; doi:10.1007/s40279-019-01088-8)
Supplement: Supplementary file 1 — Supplementary material 1 (DOCX 113 kb) [file 40279_2019_1088_MOESM1_ESM.docx]

# Electronic Supplementary Material

### Electronic Supplementary Material Appendix S1

(Immobilisation or immobilization or immobili$ or (Physical$ adj3 restrain$) or (Unweighted adj3 (limb or limb$1)) or (unweighted adj3 (leg or leg$1)) or (unweighted adj3 (arm or arm$1)))

(bed rest or bedrest$1 or (brace$1 or restraint$2) or cast or (sling or sling$1 or arm sling or arm sling$1) or (unilateral limb suspension model or unilateral lower limb suspension or unilateral lower limb suspension model or ULLS))

(Neuromuscular function or muscle function) or (muscle strength or muscle contraction or maximal voluntary contraction or maximal voluntary contraction$1 or MVC) or (Transcranial magnetic stimulation or TMS or single pulse or paired pulse or repetitive pulse) or (peripheral nerve stimulation or electrical nerve stimulation or transcutaneous nerve stimulation or percutaneous nerve stimulation or electrical stimulation or nerve stimulation) or (Electromyography or EMG) or (M-Wave or compound muscle action potential or action potential or muscle response)

### Electronic Supplementary Material Tables

Electronic Supplementary Material Table S1 MRI Characteristics

| **Study** | **MRI** | **Slice thickness (mm)** | **Slice interval (mm)** | **Number of images** |
| --- | --- | --- | --- | --- |
| Oates et al. 2010 [45] | 3T | 5 | NR | 9 mid thigh,  7 mid leg |
| Hvid et al. 2010 [35] (Suetta et al. 2009 [57], Suetta et al. 2013 [56], Hvid et al. 2011 [36]) | NR | 10 | 50 | 7 or 8 |
| Seynnes et al. 2008 [53 ], (Seynnes et al. 2008 [54]) | 0.2T | 1.56 | NR | NR |
| de Boer et al. 2007 [22] (de Boer et al. 2007 [23]) | 0.2T | 5 | 0.5 | NR |
| Hotta et al. 2011 [34] | 0.2T | 10 | 10 | 11 |
| Campbell et al. 2013 [15] | 0.2T | 3.1 | NR | NR |
| Cook et al. 2014 [19] (Cook et al. 2010 [20]) | 1.5T | 10 | 10.12 | NR |
| Tesch et al. 2004[58] | 1.5T | 10 | NR | NR |
| Seki et al. 2007[49] | 1T | NR | 2 | 4 of 21 |
| Urso et al. 2006 [59] | 1.5T | 4 | 0 | 21 |
| Yue et al. 1997 [62] | 1.5T | 5 | NR | 60 |

^MRI, magnetic resonance imaging; mm, millimetres; NR, not reported; T, tesla.^

Electronic Supplementary Material Table S2 Muscle Strength: Changes in muscle strength following immobilisation

| **Muscle group** | | **Immobilisation method** | **Study** | **Pre (SD)** | **Post (SD)** | **Unit** | **d** | **% Change** | **% Change∙d^-1^** | **Notes** |
| --- | --- | --- | --- | --- | --- | --- | --- | --- | --- | --- |
| **Lower Limb** | |  |  |  |  |  |  |  |  |  |
| Knee Extensors | | Brace/Cast | Hvid et al. 2014 (Hvid et al. 2013, Suetta et al. 2012) | 165(11) | 151(12) ^*^ | Nm | 4 | -8.00 | -2.00 | Old |
|  |  |  |  | 219(13) | 197(10) ^*^ |  |  | -10.00 | -2.50 | Young |
|  |  |  | Deschenes et al. 2008 | 200.1(13.7) | 172(13.2) ^*^ | N | 7 | -14.00 | -2.00 | Old |
|  |  |  |  | 231.3(19) | 195(19.3) ^*^ |  |  | -15.60 | -2.23 | Young |
|  |  |  | Deschenes et al. 2009a | 125(10.3) | 105.4(10.4) ^*^ | N | 7 | -16.00 | -2.29 | Male |
|  |  |  |  | 102.9(8.2) | 73(5.6) ^*^ |  |  | -28.00 | -4.00 | Female |
|  |  |  | Deschenes et al. 2009b | 207.3(49.6) | 158.7(34.2) ^*^ | Nm | 7 | -23.00 | -3.29 |  |
|  |  |  | Deschenes 2012 | 150.5(13.5) | 131.1(14.1) ^*^ | N | 7 | -13.00 | -1.86 | Male |
|  |  |  |  | 104.0(7.5) | 80.8(5.0) ^*^ |  |  | -22.00 | -3.14 | Female |
|  |  |  | Deschenes et al. 2002 | 216.5(19.3) | 169.4(21.1) ^*^ | N | 14 | -21.80 | -1.56 |  |
|  |  |  | Hvid et al. 2010 (Suetta et al. 2009, Suetta et al. 2013, Hvid et al. 2011) | 1.77(0.14) | 1.50(0.15) ^*^ | Nm/kg | 14 | -18.00 | -1.29^⬧^ | Old |
|  |  |  |  | 3.04 (0.16) | 2.56(0.14) ^*^ |  |  | -15.80 | -1.13^⬧^ | Young |
|  |  |  |  | 139(21) | 118(24) ^*^ | Nm | 14 | -19.80 | -1.41 | Old |
|  |  |  |  | 214(27) | 171(23) ^*^ |  |  | -15.70 | -1.12 | Young |
|  |  |  | Oates et al. 2010 | ~257(50) | ~196(33) ^*^ | Nm | 14 | -22.3^*^ | -1.59 |  |
|  |  |  |  |  |  |  |  |  |  |  |
|  |  | ULLS | Berg & Tesch 1996 | 305(50) | 264(49) | Nm | 10 | -13.40^*^ | -1.34 |  |
|  |  |  | de Boer et al. 2007 (de Boer et al. 2007) | 289.4(54) | 248.5 (58.58) | Nm | 14 | -14.13^*^ | -1.01 |  |
|  |  |  | Campbell et al. 2013 | 299(14) | 221(14) | NR | 21 | -26.00^*^ | -1.24 |  |
|  |  |  | Horstman et al. 2012 | 308(57) | 246(42) | Nm | 21 | -20.50^*^ | -0.98 |  |
|  |  |  | Schulze et al. 2002 | ~269.6 | ~226.2 | Nm | 21 | -18.00^*^ | -0.86 |  |
|  |  |  | Cook et al. 2014 (Cook et al. 2010) | NR | NR | N | 30 | -16.00 | -0.53 |  |
|  |  |  | Tesch et al. 2004 | ~343.81 (11.73) | ~260.27 (83.53) ^*^ | N | 35 | -24.00^*^ | -0.69 | 90 degrees |
|  |  |  |  | ~431.34 (119.38) | ~317.94 (104.74) ^*^ |  |  | -26.00^*⬧^ | -0.74^⬧^ | 120 degrees |
|  |  |  |  |  |  |  |  |  |  |  |
| Plantar Flexors | | Brace/Cast | Davies et al. 1987 | 1312(118) | 1126(177) ^*^ | N | 7 | -14.20 | -2.03 |  |
|  |  |  | White et al. 1984 | 1367(190) | 1210(283) ^*^ | N | 7 | -11.00 | -1.57 |  |
|  |  |  | Oates et al. 2010 | ~186.4(13.2) | ~140(13.6) ^*^ | Nm | 14 | -25.30^*^ | -1.81 |  |
|  |  |  |  |  |  |  |  |  |  |  |
|  |  | ULLS | Seynnes et al. 2008, Seynnes et al. 2008 | 165.7(9.6) | 149.4(9.4) ^*^ | Nm | 14 | -10.00^*^ | -0.71 |  |
|  |  |  | Hotta et al. 2011 | ~142.36 (29.45) | ~134.41 (35.34) | Nm | 20 | -5.58 | -0.28 |  |
|  |  |  | Horstman et al. 2012 | 242(54) | 211(36) | Nm | 21 | -12.10^*^ | -0.58 |  |
|  |  |  | Schulze et al. 2002 | ~186.3 | ~158.3 | Nm | 21 | -17.00^*^ | -0.81 |  |
|  |  |  | Seynnes et al. 2010 | 210(18) | 178(13) ^*^ | Nm | 24 | -15.20^*^ | -0.63 |  |
|  |  |  | Cook et al. 2014 (Cook et al. 2010) | 279.6(124.6) | NR | N | 30 | -26.70^*^ | -0.89 |  |
| **Ankle** | |  |  |  |  |  |  |  |  |  |
| Plantar Flexors | | Brace/Cast | Lundbye-Jensen & Nielsen 2008 | 69.2  [69.1(21.2), 69.3(19.8)] | 59(20) | Nm | 14 | -15.00^*^ | -1.07 |  |
|  |  |  | Gondin et al. 2004 | 124(6) | 103(7) | Nm | 14 | -17.00^*^ | -1.21 |  |
|  |  |  |  |  |  |  |  |  |  |  |
| Dorsi Flexors | | Brace/Cast | Lundbye-Jensen & Nielsen 2008 | 43.9  [44.1 (9.2), 43.7 (11)] | 33.6(8.1) | Nm | 14 | -23.00^*^ | -1.64 |  |
| **Upper Limb** | |  |  |  |  |  |  |  |  |  |
| Elbow Flexors | | Brace/Cast | Karolczak et al. 2009 | 74(10) | 62(11) ^*^ | Nm | 14 | -16.22 | -1.16 |  |
|  |  |  | Vaughan et al. 1989 | 28.22 | 24.62 (8.36) ^*^ | Kg | 14 | -12.76 | -0.91 |  |
|  |  |  | Yue et al. 1997 | 199(58) | 136(51) ^*^ | N | 28 | -35.00 | -1.25 |  |
|  |  |  |  |  |  |  |  |  |  |  |
|  |  | Sling | Magnus et al. 2010 | NR | NR | NR | 27.8 | 4.00 | 0.14 |  |
|  |  |  | Pearce et al. 2013 | 198.0(8.1) | 186.6(10) ^*^ | N | 21 | -5.80 | -0.28 |  |
|  |  |  |  |  |  |  |  |  |  |  |
| Elbow Extensors | | Brace/Cast | Karolczak et al. 2009 | 60(10) | 49(6) ^*^ | Nm | 14 | -18.30 | -1.31 |  |
|  |  |  | Vaughan et al. 1989 | 18.12 | 16.39 (8.01) | Kg | 14 | -9.55 | -0.68 |  |
|  |  |  | Yue et al. 1997 | 159(55) | 115(59) ^*^ | N | 28 | -30.00 | -1.07 |  |
|  |  | Sling | Magnus et al. 2010 | NR | NR | NR | 27.8 | -6.10 | -0.22 |  |
|  |  |  |  |  |  |  |  |  |  |  |
| Forearm Flexors | | Brace/Cast | Farthing et al. 2009 | ~12.2 | ~10.34 | Nm | 21 | -14.70^*^ | -0.70 |  |
|  | |  | Farthing et al. 2011 | ~34.5 | ~30.8 | Kg | 21 | -10.72^*^ | -0.51 |  |
|  |  |  |  |  |  |  |  |  |  |  |
| Wrist Flexors | | Brace/Cast | Clark et al. 2008 | ~8.46 | ~6.69 | Nm | 7 | -27.00^*^ | -3.86 |  |
|  |  |  | Lundbye-Jensen & Nielsen 2008 | 14.78  [14.59 (2.22), 14.98 (1.95)] | 11.14(1.4) | Nm | 7 | -24.68^*^ | -3.53 |  |
|  |  |  | Clark et al. 2010 | ~20.6 | ~11.1 | Nm | 21 | -43.20^*^ | -2.06 |  |
|  |  |  | Clark et al. 2014 | 21.8(1.8) | ~11.6 | Nm | 28 | -45.10 | -1.61 |  |
|  |  |  |  |  |  |  |  |  |  |  |
| Wrist Extensors | | Brace/Cast | Lundbye-Jensen & Nielsen 2008 | 8.38  [8.25 (1.26), 8.51 (1.13)] | 6.34(0.69) | Nm | 7 | -24.34^*^ | -3.48 |  |
|  |  |  |  |  |  |  |  |  |  |  |
| Thumb Abduction | | Brace/Cast | Sale et al. 1982 | ~48(8) | ~26(6) | Nm | 35 | -45.83 | -1.31 | Group A, n=3 |
|  |  |  |  | ~46.88 (5) | ~20.63(3.13) |  |  | -56.00 | -1.60 | Group B, n=3 |
|  |  |  |  |  |  |  |  |  |  |  |
| Thumb Adduction | | Brace/Cast | Urso et al. 2006 | 71.74 (5.2) | ~56 | N | 14 | -22.00 | -1.57 | Old |
|  |  |  |  | 103.6 (2.9) | ~81 |  |  | -22.00 | -1.57 | Young |
|  |  |  |  |  |  |  |  |  |  |  |
| First Dorsal Interosseous | | Brace/Cast | Inada et al. 2016 | 37.8(6.1) | 32.8(6.9) | N | 0.5 | -13.23 | -26.46 |  |
|  |  |  | Ngomo et al. 2012 | NR | NR | NR | 4 | -33.00 | -8.25 |  |
|  |  |  | Fuglevand et al. 1995 | 33.6(8.2) | 35.0 (8.1) | N | 7 | 4.17 | 0.60 |  |
|  |  |  | Seki et al. 2007 | 36.46(13.9) | 28.81(10.19) ^*^ | N | 7 | -21.00 | -3.00 |  |
|  |  |  | Seki et al.2001 | 38.9(9.1) | 34.1(5.9) ^*^ | N | 21 | -12.34 | -0.59 |  |

^d, days; NR, not reported; ULLS, unilateral limb suspension; [ ], multiple pre visits; *, reported by authors as significant; ⬧ , not used in analysis; ~, approximately stated or mean value given.^

Electronic Supplementary Material Table S3 Muscle Size: Changes in muscle size following immobilisation

| **Location** | **Immobilisation method** | **Study** | **Acquisition** | **Area** | **Pre (SD)** | **Post (SD)** | **d** | **% Change** | **% Change∙d^-1^** | **Notes** |
| --- | --- | --- | --- | --- | --- | --- | --- | --- | --- | --- |
| **Lower Limb** |  |  |  |  |  |  |  |  |  |  |
| Muscle Fibre | Brace/Cast | Hvid et al. 2014 | VL M-fibre CSA | Type I | ~4666.7 (254) | ~4286(254) | 4 | -7.10 ^*^ | -1.78 | Old |
|  |  |  |  | Type II | ~3924.2 (242.4) | ~3409.1 (212.1) ^*^ | 4 | -10.90 ^*^ | -2.73 | Old |
|  |  |  |  | Type I | ~5063.5 (142.9) | ~4634.9 (238.1) ^*^ | 4 | -8.10 ^*^ | -2.03 | Young |
|  |  |  |  | Type II | ~5575.8 (374) | ~4818.2 (409.1) ^*^ | 4 | -12.60 ^*^ | -3.15 | Young |
|  |  | Deschenes et al. 2002 | M-fibre CSA, 10 μm transverse sections | Type I | 4715(340) | 4548(467) | 14 | -3.54 | -0.25 |  |
|  |  |  |  | Type IIA | 4840(448) | 4587(390) | 14 | -5.23 | -0.37 |  |
|  |  |  |  | Type IIB | 4307(412) | 4052(381) | 14 | -5.92 | -0.42 |  |
|  |  |  |  | Combined | 4789(392) | 4570(428) | 14 | -4.57 | -0.33 |  |
|  |  | Hvid et al. 2010 | M-fibre CSA | Type I | 5301(497) | 4830.3(587) | 14 | -8.89 | -0.64 | Old |
|  |  |  |  | Type II | 5029(634) | 4269(545) | 14 | -13.20 | -0.94 | Old |
|  |  |  |  | Type IIx Old | 3715(444) | 2924(200) | 14 | -18.80 | -1.34 | Old |
|  |  |  |  | Type I | 5180(480) | 4440(500) ^*^ | 14 | -15.20 | -1.09 | Young |
|  |  |  |  | Type IIa | 6073(448) | 4537(480) ^*^ | 14 | -25.30 | -1.81 | Young |
|  |  |  |  | Type IIx | 5458(344) | 3891(441) ^*^ | 14 | -29.70 | -2.12 | Young |
| Above Knee | Brace/Cast | Deschenes et al. 2012 | DXA | Lean mass thigh | 6410(248.7) | 6229.7 (219.5) | 7 | -2.81 | -0.40 | Male |
|  |  |  |  | Lean mass thigh | 4715.9 (204.1) | 4641.8 (181.8) ^*^ | 7 | -1.57 | -0.22 | Female |
|  |  | Oates et al. 2010 | MRI | Thigh | ~146.3(25.3) | ~136.8(23.3) ^*^ | 14 | -6.20 | -0.44^⬧^ |  |
|  |  |  |  | Quadriceps Femoris | NR | NR | 14 | -7.60 ^*^ | -0.54 |  |
|  |  | Suetta et al. 2009 | MRI | Qvol | 1841.3(62.2) | 1676.9(47.3) ^*^ | 14 | -8.90 | -0.64 | Young |
|  |  |  |  | QVol | 1633.1(46.3) | 1545(39.7) ^*^ | 14 | -5.20 | -0.37 | Old |
|  | ULLS | de Boer et al. 2007 (de Boer et al. 2007) | MRI | Quadriceps | 56.27(9.48) | 53.35(8.86) ^*^ | 14 | -5.19 ^*^ | -0.37 |  |
|  |  |  |  | Vastus Medialis | 20.39(3.61) | 19.39(3.29) ^*^ | 14 | -4.90 | -0.35^⬧^ |  |
|  |  |  |  | Rectus Femoris | 2.75(1.04) | 2.64(0.98) ^*^ | 14 | -4.00 | -0.29^⬧^ |  |
|  |  |  |  | Vastus Lateralis | 16.50(3.63) | 15.94(3.48) ^*^ | 14 | -3.39 | -0.24^⬧^ |  |
|  |  |  |  | Vastus Intermedius | 16.63(2.70) | 15.37(2.47) ^*^ | 14 | -7.58 | -0.54^⬧^ |  |
|  |  | Campbell et al. 2013 | MRI | Quadriceps Femoris | 1983(116) | 1771(79) ^*^ | 21 | -10.00 | -0.48 |  |
|  |  |  |  | Vastus Lateralis | 663(32) | 585(16) ^*^ | 21 | -12.00 | -0.57^⬧^ |  |
|  |  |  |  | Vastus Medialis | 493(10) | 449(23) ^*^ | 21 | -9.00 | -0.43^⬧^ |  |
|  |  |  |  | Vastus Intermedius | 533(50) | 466(40) ^*^ | 21 | -12.57 | -0.60^⬧^ |  |
|  |  |  |  | Rectus Femoris | NR | NR | 21 | -5.00 | -0.24^⬧^ |  |
|  |  | Schulze et al. 2002 | CT | Thigh | ~155.7(32.8) | ~145.1(34.4) | 21 | -7.00 ^*^ | -0.33 |  |
|  |  | Cook et al. 2014/2010 | MRI | Knee Extensors | 69.3(33.9) | NR | 30 | -7.50 | -0.25 |  |
|  |  | Tesch, Trieschmann, Ekberg 2004 | MRI | Vastus Lateralis | 376(95) | 341(98) | 35 | -9.30 ^*^ | -0.27^⬧^ |  |
|  |  |  |  | Vastus Intermedius | 340(67) | 310(61) | 35 | -8.80 ^*^ | -0.25^⬧^ |  |
|  |  |  |  | Vastus Medialis | 224(60) | 197(51) | 35 | -12.10 ^*^ | -0.35^⬧^ |  |
|  |  |  |  | Rectus Femoris | 101(23) | 101(25) | 35 | 0.00 | 0.00^⬧^ |  |
|  |  |  |  | Quadriceps | 1041(235) | 949(225) | 35 | -8.80 ^*^ | -0.25 |  |
| Below Knee | Brace/Cast | White et al. 1984 | Anthropometry | Lower Leg (inc bone) | ~98.5(5.94) | ~93.8(6.51) | 7 | -5.00 ^*^ | -0.71 |  |
|  |  | Davies et al. 1987 | Anthropometry | Lower Leg (inc bone) | ~66.23 | ~64.22 | 7 | -3.00 | -0.43 |  |
|  |  | Oates et al. 2010 | MRI | Gastrocnemius | NR | NR | 14 | -9.40 ^*^ | -0.67 |  |
|  |  |  |  | Soleus | NR | NR | 14 | -6.80 ^*^ | -0.49 |  |
|  | ULLS | Seynnes et al. 2008, Seynnes et al. 2008 | MRI | Soleus Volume | 527.8(33.4) | 503.2(32.1) ^*^ | 14 | -5.00 | -0.36 |  |
|  |  |  |  | Soleus CSA | 28.3 (1.7) | 26.3(1.8) ^*^ | 14 | -7.00 | -0.50^⬧^ |  |
|  |  |  |  | Gastrocnemius Lateralis Volume | 121.3(12) | 114.4(10.1) ^*^ | 14 | -5.00 | -0.36 |  |
|  |  |  |  | Gastrocnemius Lateralis CSA | 9.8(0.8) | 8.9(0.7) ^*^ | 14 | -9.20 | -0.66^⬧^ |  |
|  |  |  |  | Gastrocnemius Medialis Volume | 216.6(20.7) | 203.4(19.1) ^*^ | 14 | -6.00 | -0.43 |  |
|  |  |  |  | Gastrocnemius Medialis CSA | 14.4(1.4) | 13.1(1.3) ^*^ | 14 | -9.00 | -0.64^⬧^ |  |
|  |  | Hotta et al. 2011 | MRI | Calf | ~37.12 (8.94) | ~33.88 (19.52) | 20 | -8.73 | -0.44 |  |
|  |  | Schulze et al. 2002 | CT | Lower Leg | ~77.6(12.6) | ~70.5(10.4) | 21 | -7.00 | -0.33 |  |
|  |  | Cook et al. 2014/2010 | MRI | Plantar Flexors | 36.6(11.5) | NR | 30 | -8.50 | -0.28 |  |
| **Upper Limb** | Brace/Cast | Seki et al. 2007 | MRI | First Dorsal Interosseous | 247.6(18.8) | 245.3(15.8) | 7 | -1.00 | -0.14 |  |
|  |  | Urso et al. 2006 | MRI | Adductor Pollicis | ~12.94(0.44) | ~11.71(0.33) ^*^ | 14 | -9.50 | -0.68 | Old |
|  |  |  |  | Adductor Pollicis | ~13.69(0.31) | ~13.14(0.34) | 14 | -4.00 | -0.29 | Young |
|  |  | Farthing et al. 2011 | Ultrasound | Flexor Carpi Ulnaris & Flexor Digitorum Superficialis | 3.6 | 3.54(0.06) | 21 | -1.70 | -0.08 |  |
|  |  | Farthing et al. 2009 | Ultrasound | Flexor Carpi Ulnaris & Flexor Digitorum Superficialis | ~3.47(0.18) | ~3.32(0.17) | 21 | -4.30 ^*^ | -0.20 |  |
|  |  | Yue et al. 1997 | MRI | Elbow flexors CSA | 19.8(7.4) | 17.6(5.6) ^*^ | 28 | -11.11 | -0.40^⬧^ |  |
|  |  |  |  | Triceps Brachii CSA | 23.2(7.2) | 22.3(7.3) | 28 | -3.88 | -0.14^⬧^ |  |
|  |  |  |  | Elbow flexors Volume | 317(142) | 281(111) ^*^ | 28 | -11.36 | -0.41 |  |
|  |  |  |  | Triceps Brachii Volume | 359(141) | 353(137) | 28 | -1.67 | -0.06 |  |
|  | Sling | Pearce et al. 2013 | Ultrasound | Biceps Brachii & Brachialis | 25.1(1.3) | 23.6(1.5) ^*^ | 21 | -6.00 | -0.29 |  |
|  |  | Magnus et al. 2010 | Ultrasound | Biceps Brachii | NR | NR | 28 | -2.80 | -0.10 |  |
|  |  |  |  | Triceps Brachii | NR | NR | 28 | -5.20 | -0.19 |  |

^CSA, cross sectional area; CT, computed tomography; d, days; DXA, duel x-ray absorptiometry; MRI, magnetic resonance imaging; NR, not reported; ULLS, unilateral limb suspension; *, reported by authors as significant; ⬧ , not used in analysis; ~, approximately stated or mean value given.^

Electronic Supplementary Material Table S4 Muscle Contractility: Changes in resting twitch force following immobilisation

| **Muscle group** | **Immobilisation method** | **Study** | **Stimulation location** | **Pre (SD)** | **Post (SD)** | **d** | **Units** | **% Change** | **% Change∙d^-1^** | **Notes** |
| --- | --- | --- | --- | --- | --- | --- | --- | --- | --- | --- |
| **Lower Limb** |  |  |  |  |  |  |  |  |  |  |
| Knee Extensors | Brace/Cast | Hvid et al. 2010 (Suetta et al. 2009, Suetta et al. 2013, Hvid et al. 2011) | Muscle | 47.72 (3.81) | 35.79 (2.07) | 14 | Nm | -22.20 | -1.59 | Old |
|  |  |  |  | 27.40 (1.95) | 19.80 (3.65) | 14 |  | -27.70 | -1.98 | Young |
|  | ULLS | Campbell et al. 2013 | Nerve | 66(4) | 58(6) | 21 |  | -12.00 | -0.57 |  |
|  |  | Horstman et al. 2012 | Muscle | 76(12) | 68(15) | 21 | Nm | -11.30 | -0.54 | Triplet |
|  |  | Cook et al. 2014 (Cook et al. 2010) | Nerve | 200(55) | 210(67) | 30 | N | 6.00 | 0.20 |  |
|  |  |  |  | 319(94) | 312(82) |  |  | -2.19 | -0.07 | Doublet |
| Plantar Flexors | Brace/Cast | Davies et al. 1987 | Muscle | 128(30) | 143(48) | 7 | N | 10.50 | 1.50 |  |
|  |  | White et al. 1984 | Muscle | 142(28) | 147(32) | 7 | N | 3.00 | 0.43 |  |
|  | ULLS | Seynnes et al. 2010 | Nerve | 19.1(1.8) | 22.7(2.0) | 24 | Nm | 21.00 | 0.88 |  |
|  |  | Cook et al. 2014 (Cook et al. 2010) | Nerve | 35(17) | 34(17) | 30 | N | -2.86 | -0.10 |  |
|  |  |  |  | 68(33) | 60(29) |  |  | -11.76 | -0.39^⬧^ | Doublet |
| **Ankle** |  |  |  |  |  |  |  |  |  |  |
| Plantar Flexors | Brace/Cast | Gondin 2004 | Nerve | 20.1(1.3) | 22.2(2.0) | 14 | Nm | 10.45 | 0.75 |  |
|  |  |  |  | 36.2(3) | 38.9(3.6) | 14 |  | 7.46 | 0.53^⬧^ | Doublet |
|  |  | Lundbye-Jensen & Nielsen 2008 | Nerve | 5.29 [5.34 (2.83), 5.24 (2.83)] | 8.29(4.6) | 14 | Nm | 56.71 | 4.05 |  |
| **Upper Limb** |  |  |  |  |  |  |  |  |  |  |
| Elbow Flexors | Brace/Cast | Yue et al. 1997 | Muscle | 17.7(3.5) | 21.7(4.9) ^*^ | 28 | N | 22.60 | 0.81 | Biceps brachii short head |
|  |  |  |  | 10.2(7.7) | 11.5(8.7) |  | N | 12.75 | 0.46^⬧^ | Brachioradialis |
|  | Brace/Cast | Lundbye-Jensen & Nielsen 2008 | Nerve | 0.8 [0.79(0.49),0.80(0.11)] | 0.77 (0.17) | 7 | Nm | -3.75 | -0.54 |  |
|  |  | Clark et al. 2008 | Nerve | 1.72(0.27) | 1.56(0.3) | 21 | Nm | -9.30 | -0.44^⬧^ |  |
| Thumb Abduction | Brace/Cast | Sale 1982 | Nerve | ~15.77(2.44) | ~22.44 (3.74) | 35 | N | 42.30 | 1.21 | Group A, n=6 |
|  |  |  |  | ~26.25(4.06) | ~27.5 (5.31) |  |  | 3.81 | 0.11 | Group B, n=4 |
| First Dorsal Interosseous | Brace/Cast | Inada 2016 | Nerve | 0.86(0.43) | 1.16 (0.49) | 0.5 | N | 34.88 | 69.76 |  |
|  |  | Fuglevand 1995 | Nerve | 3.2 (0.8) | 4.1(1.1) | 7 | N | 28.13 | 4.02 | n=9 |
|  |  | Seki 2001 | Muscle | 2.4(1.3) | 2.9(0.9) | 21 | N | 20.83 | 0.99 |  |

^d, days; N, newtons; Nm, newton metre; [ ], multiple pre visits; *, reported by authors as significant; ⬧ , not used in analysis; ~, approximately stated or mean value given.^

Electronic Supplementary Material Table S5 Muscle Contractility: Changes in force development following immobilisation

| **Muscle group** | **Immobilisation method** | **Study** | **Measure** | **Pre (SD)** | **Post (SD)** | **Units** | **d** | **% Change** | **% Change∙d^-1^** | **Notes** |
| --- | --- | --- | --- | --- | --- | --- | --- | --- | --- | --- |
| **Lower Limb** | |  |  |  |  |  |  |  |  |  |
| Knee Extensors | Brace/Cast | Hvid et al. 2014 | rRFD100 | 534(25) | 441(17) | %MVC.s | 4 | -17.40 | -4.35 | Old |
|  |  |  |  | 558(35) | 528(28) |  |  | -5.40 | -1.35 | Young |
|  |  | Hvid et al. 2010 | rRFD100 | 480(62) | 373(28) ^*^ | %MVC.s | 14 | -22.30 | -1.59^⬧^ | Old |
|  |  |  |  | 456(39) | 436(27) ^*^ |  |  | -4.40 | -0.31^⬧^ | Young |
|  |  |  | rRFD50 | 548(91) | 336(34) ^*^ | %MVC.s |  | -38.69 | -2.76^⬧^ | Old |
|  |  |  | rRFD50 | 527(68) | 522(49) ^*^ |  |  | -0.95 | -0.07^⬧^ | Young |
|  |  |  | Impulse50 | 12.7 (2.1) | 7.6(1.1) ^*^ | Nm.s.kg^-1^ |  | -40.16 | -2.87^⬧^ | Old |
|  |  |  | Impulse50 | 22(2.7) | 19.1(1.9) |  |  | -13.18 | -0.94^⬧^ | Young |
|  |  |  | Impulse100 | 50.7 (7.8) | 32.2(4.7) ^*^ | Nm.s.kg^-1^ |  | -36.29 | -2.59^⬧^ | Old |
|  |  |  | Impulse100 | 84.2 (9.5) | 68.9(6.4) |  |  | -18.17 | -1.30^⬧^ | Young |
|  |  |  | RFD50 | 9.6(1.8) | 5.4(0.9) ^*^ | Nm.s^-1^.kg^-1^ |  | -43.75 | -3.13^⬧^ | Old |
|  |  |  | RFD50 | 16.2 (2.3) | 13.3(1.5) |  |  | -17.90 | -1.28^⬧^ | young |
|  |  |  | RFD100 | 8.4(1.3) | 5.7(0.8) ^*^ | Nm.s^-1^.kg^-1^ |  | -32.14 | -2.30^⬧^ | Old |
|  |  |  | RFD100 | 14.0 (1.4) | 11.1(0.9) ^*^ |  |  | -20.71 | -1.48^⬧^ | young |
|  |  | Suetta 2009 | TPT | 88(2) | 88(2) | Ms | 14 | 0.00 | 0.00 | young |
|  |  |  | RFD 0-30ms | 1375 (99) | 1053(72) ^*^ | Nm.s-^1^ |  | -21.70 | -1.55^⬧^ |  |
|  |  |  | RFD 0-50ms | 1778 (126) | 1361(90) ^*^ | Nm.s^-1^ |  | -21.50 | -1.54^⬧^ |  |
|  |  |  | TPT | 89(2) | 90(2) | Ms |  | 1.12 | 0.08 | Old |
|  |  |  | RFD 0-30ms | 738(48) | 546(97) ^*^ | Nm.s^-1^ |  | -30.70 | -2.19^⬧^ |  |
|  |  |  | RFD 0-50ms | 980(66) | 734(131) ^*^ | Nm.s^-1^ |  | -30.40 | -2.17^⬧^ |  |
|  | ULLS | de Boer et al. 2007 (de Boer et al. 2007) | RTD | 1206.1 (464.3) | 697.8 (266.5) ^*^ | Nm.s^-1^ | 14 | -42.14 ^*^ | -3.01 |  |
|  |  | Campbell et al. 2013 | TPT | 58(2) | 62(2) | NR | 21 | 6.90 | 0.33 |  |
|  |  | Horstman et al. 2012 | Voluntary MRTD | 2.68 (0.66) | 2.11(0.33) | kNm s^-1^ | 21 | -15.60 | -0.74^⬧^ |  |
|  |  |  | Evoked MRTD | 2.65 (0.75) | 2.15(0.52) ^*^ |  |  | -17.00 ^*^ | -0.81 |  |
|  |  | Cook et al. 2014 (Cook et al 2010) | RFD | NR | +1.3(3.7) | Nm.s^-1^ | 30 | NR | NR |  |
| Plantar Flexors | Brace/Cast | Davies et al. 1987 | TPT | 130(10) | 144(21) ^*^ | ms | 7 | 10.80 | 1.54 |  |
|  |  | White et al. 1984 | TPT | 126(10) | 143(20) ^*^ | ms | 7 | 13.50 | 1.93 |  |
|  | ULLS | Horstman et al. 2012 | Voluntary MRTD | 0.92 (0.17) | 0.76(0.13) | kNm s^-1^ | 21 | -16.90 | -0.80^⬧^ |  |
|  |  | Seynnes et al. 2010 | MRTD | 321(33) | 314(30) | Nm.s^-1^ | 24 | -2.20 | -0.09 |  |
|  |  | Cook et al. 2014 (Cook et al. 2010) | RFD | NR | +0.04(0.14) | N.ms^-2^ | 30 | NR | NR |  |
| **Ankle** | |  |  |  |  |  |  |  |  |  |
| Plantar Flexors | Brace/Cast | Gondin 2004 | TPT | 136.2(2) | 159.3(6.1) ^*^ | ms | 14 | 16.96 | 1.21 |  |
|  |  |  | TPT | 162(4.3) | 184.6(9.3) |  |  | -13.95 | -1.00^⬧^ | Doublet |
|  |  | Lundbye-Jensen & Nielsen 2008 | CT | 113.95 [116.5(21),111.4(17)] | 114.7(19) | ms | 14 | 0.66 | 0.05 |  |
| **Upper Limb** | |  |  |  |  |  |  |  |  |  |
| Elbow Flexors | Brace/Cast | Vaughan 1989 | MT | 148.26 | 161.55 (19.80) | ms | 14 | 8.96 | 0.64 |  |
|  |  |  |  |  |  |  |  |  |  |  |
|  |  | Yue et al. 1997 | CT | 71.6 (10.1) | 70.8(7.8) | ms | 28 | -1.12 | -0.04 | Biceps brachii short head |
|  |  |  | CT | 42.8 (11.9) | 47.4(13.1) |  |  | 10.75 | 0.38 | Brachioradialis |
| Elbow Extensors | Brace/Cast | Vaughan 1989 | MT | 154.075 | 165.20 (20.22) | ms | 14 | 7.22 | 0.52 |  |
| Wrist Flexors | Brace/Cast | Lundbye-Jensen & Nielsen 2008 | TPT | 65.65 [65.2(7),66.1(8] | 65.1(7) | ms | 7 | -0.84 | -0.12 |  |
|  |  |  |  |  |  |  |  |  |  |  |
|  |  | Clark et al. 2008 | RFD | ~20 | ~15.65 | %Peak Force/ ms | 21 | -21.75 | -1.04^⬧^ |  |
| Thenar Muscles | Brace/Cast | Sale 1982 | CT | ~51.56 (3.75) | ~57.19 (5.63) | ms | 35 | 10.92 | 0.31 | Group 1, n=4 |
|  |  |  | CT | ~49(2.2) | ~56.1(3.17) |  |  | 14.49 | 0.41 | Group 2, n=6 |
| First Dorsal Interosseous | Brace/Cast | Fuglevand 1995 | CT | 65(14) | 70(13) | ms | 7 | 7.69 | 1.10 | n=9 |
|  |  | Seki 2001 | CT | ~63.91 (10.48) | ~67.65 (9.06) | ms | 21 | 5.85 | 0.28 |  |

^CT, contraction time; d, days; k, kilo; MRTD, maximum rate of torque development; ms, milliseconds; MT, movement time; MVC, maximal voluntary contraction; N, newtons; Nm, newton metres; r, relative; RFD, rate of force development; RTD, rate of torque development; TPT, time to peak twitch; ULLS, unilateral lower limb suspension; [ ], multiple pre visits; *, reported by authors as significant; ⬧ , not used in analysis; ~, approximately stated or mean value given.^

Electronic Supplementary Material Table S6 Muscle Contractility: Changes in force relaxation following immobilisation

| **Muscle group** | **Immobilisation method** | **Study** | **Measure** | **Unit** | **Pre (SD)** | **Post (SD)** | **d** | **% Change** | **% Change∙d^-1^** | **Notes** |
| --- | --- | --- | --- | --- | --- | --- | --- | --- | --- | --- |
| **Lower Limb** |  |  |  |  |  |  |  |  |  |  |
| Knee Extensors | ULLS | Campbell et al. 2013 | 0.5_RT_ |  | 71(6) | 78(9) | 21 | 9.86 | 0.47 |  |
|  |  | Cook et al. 2014 (Cook et al. 2010) | RFR |  | NR | 0.01(6.2) | 30 | -- | -- |  |
| Plantar Flexors | Brace/Cast | Davies et al. 1987 | 0.5_RT_ | ms | 109(19) | 128(28) ^*^ | 7 | 17.40 ^*^ | 2.49 |  |
|  |  | White et al. 1984 | 0.5_RT_ | ms | 88(7) | 107(12) ^*^ | 7 | 21.60 ^*^ | 3.09 |  |
|  | ULLS | Seynnes et al. 2010 | MRTR |  | 202(15) | 198(21) | 24 | -1.98 | -0.08 |  |
|  |  | Cook et al. 2014 (Cook et al. 2010) | RFR |  | NR | -0.14(0.44) | 30 | -- | -- |  |
| **Ankle** |  |  |  |  |  |  |  |  |  |  |
| Plantar Flexors | Brace/ Cast | Lundbye-Jensen & Nielsen 2008 | 0.5_RT_ | ms | 84.5 [83(21), 86(23)] | 101.6(16) | 14 | 20.24 | 1.45 |  |
|  |  | Gondin 2004 | 0.5_RT_ | ms | 99.3(3.9) | 111.2(6.4) | 14 | 11.98 | 0.86 |  |
| **Upper Limb** |  |  |  |  |  |  |  |  |  |  |
| Wrist Flexors | Brace/ Cast | Lundbye-Jensen & Nielsen 2008b | 0.5_RT_ | ms | 58.3 [57.2(4), 59.4(8)] | 59.2(6) | 7 | 1.54 | 0.22 |  |
|  |  | Clark et al. 2008 | RFR | %peak force/ms | ~14.74(1.23) | ~11.05(0.88) | 21 | -25.03 | -1.19^⬧^ |  |
| Thenar Muscles | Brace/ Cast | Sale 1982 | 0.5_RT_ | ms | ~55.25(4.25) | ~60.75(5.75) | 35 | 9.95 | 0.28 | Group A, n=6 |
|  |  |  |  |  | ~48.28(5.16) | ~51.56(2.81) |  | 6.79 | 0.19 | Group B, n=4 |
| First Dorsal Interosseous | Brace/Cast | Seki 2001 | 0.5_RT_ | ms | ~55.8(6.8) | ~59.2(6.4) | 21 | 6.09 | 0.29 |  |

^0.5RT, half relaxation time; d, days; MRTR, maximum rate of torque relaxation; ms, millisecond; NR, not reported; RFR, rate of force relaxation; ULLS, unilateral lower limb suspension; [ ], multiple pre visits; *, reported by authors as significant; ⬧ , not used in analysis; ~, approximately stated or mean value given.^

Electronic Supplementary Material Table S7 Central Motor Drive: Changes in central motor drive following immobilisation

| **Muscle group** | **Immobilisation method** | **Study** | **Measurement method** | **Pre (SD)** | **Post (SD)** | **d** | **% Change** | **% Change∙d^-1^** | **Notes** |
| --- | --- | --- | --- | --- | --- | --- | --- | --- | --- |
| **Lower Limb** |  |  |  |  |  |  |  |  |  |
| Knee extensors | Brace/Cast | Suetta et al. 2009 | CAR | 91.6(1.6) | 90.6(2.8) | 14 | -1.10 | -0.08 | Young |
|  |  |  | CAR | 88.6(1.6) | 80.2(2.8) | 14 | -9.90 ^*^ | -0.71 | Old |
|  | ULLS | de Boer et al. 2007 (de Boer et al. 2007) | CAR | 98.4(0.7) | 97.8(1.3) | 14 | -0.61 | -0.04 |  |
|  |  | Oates et al. 2010 | %MUA | 94.6(2) | 95.2(0.8) | 14 | 0.63 | 0.05 |  |
|  |  | Campbell et al. 2013 | VA | 87(3.5) | 83(3.5) | 21 | -5.10 | -0.24 | Doublets |
|  |  | Horstman et al. 2012 | VA | 88.3(6.8) | 84.7(9.1) | 21 | -4.10 | -0.20 | Triplets, n=6 |
|  |  | Cook et al. 2014 (Cook et al. 2010) | CAR | ~91 | ~86 | 30 | -5.49 | -0.18 |  |
| Plantar Flexors | ULLS | Oates et al. 2010 | %MUA | 95.4(1.0) | 95.7(1.5) | 14 | 0.31 | 0.02 |  |
|  |  | Hotta et al. 2011 | VA | ~93.29(5.45) | ~89.69(8.39) | 20 | -3.86 | -0.19 |  |
|  |  | Seynnes et al. 2010 | AC | 97.9(0.6) | 96.5(1) | 24 | -1.43 | -0.06 |  |
|  |  | Cook et al. 2014 (Cook et al 2010) | CAR | ~92 | ~85 | 30 | -7.61 | -0.25 |  |
| **Ankle** |  |  |  |  |  |  |  |  |  |
| Plantar Flexors | Brace/Cast | Gondin et al. 2004 | VA Resting | 94.2(3.3) | 86.9(4.4) | 14 | -8(4) ^*^ | -0.57 | Doublet |
|  |  |  | VA Potentiated | 94.4(3.1) | 86.8(4.5) | 14 | -8(4) | -0.57^⬧^ | Doublet |
|  |  |  | CAR | 98.1(1.1) | 94.3(1.9) | 14 | -4(1) | -0.29^⬧^ |  |
| **Upper Limb** |  |  |  |  |  |  |  |  |  |
| Elbow Flexors | Sling | Magnus et al. 2010 | Activation | 91.5(4.6) | 89.7(9.1) | 27.8 | -2.00 | -0.07 |  |
| Elbow Extensors | Sling | Magnus et al. 2010 | Activation | 87.1(7.0) | 90.4(8.3) | 27.8 | 3.80 | 0.14 |  |
| Wrist Flexors | Brace/Cast | Clark et al. 2008 | CAR | 85.6(3.4) | 66.7(6.8) | 21 | -22.10 | -1.05^⬧^ |  |
|  |  | Clark et al. 2010 | CAR | 97.5(2.4) | 73.2(8.3) | 21 | -24.90 ^*^ | -1.19 |  |
|  |  | Clark et al. 2014 | VA | 96.2(1.8) | 73.9 | 28 | -23.2(5.8) | -0.83 |  |

^AC, activation capacity; CAR, central activation ratio; d, days; MUA, motor unit activation; ULLS, unilateral lower limb suspension; VA, voluntary activation; [ ], multiple pre visits; *, reported by authors as significant; ⬧ , not used in analysis; ~, approximately stated or mean value given.^

Electronic Supplementary Material Table S8 Muscle and Corticospinal Excitability: Changes in Hoffman reflex following immobilisation

| **Muscle group** | **Immobilisation method** | **Study** | **Pre (SD)** | **Post (SD)** | **Units** | **d** | **% Change** | **% Change∙d^-1^** |
| --- | --- | --- | --- | --- | --- | --- | --- | --- |
| Lower Limb |  |  |  |  |  |  |  |  |
| Plantar Flexors | ULLS | Seynnes et al. 2008, Seynnes et al. 2008 | NR | NR |  | 14 | 35.00 | 2.50 |
|  |  | Seynnes et al. 2010 | 0.60(0.14) | 0.75(0.10) | %/Max | 24 | 25.00 | 1.04 |
| Upper Limb |  |  |  |  |  |  |  |  |
| Wrist Flexors | Brace/Cast | Clark et al. 2008 | 38.2(4.9) | 47.3(7.7) | Hmax/Mmax | 7 | 23.82 | 3.40 |
|  |  | Lundbye-Jensen & Nielsen 2008 | 20.6 [21.5(5.5), 19.7(6.6)] | 36.3(7.2) | Hmax/Mmax | 7 | 76.21 | 10.89 |
|  |  | Clark et al. 2010 | 25.0(5.9) | 44.6(7.6) | Hmax/Mmax | 21 | 78.40 | 3.73 |

^d, days; Hmax, maximal amplitude of Hoffman reflex; Mmax, maximal amplitude of Mwave; NR, not reported; ULLS, unilateral lower limb suspension; [ ], multiple pre visits; ~, approximately stated or mean value given.^

Electronic Supplementary Material Table S9 Muscle and Corticospinal Excitability: Changes in motor evoked potential following immobilisation

| **Muscle group** | **Immobilisation method** | **Study** | **Pre (SD)** | **Post (SD)** | **Units** | **d** | **% Change** | **% Change∙d^-1^** |
| --- | --- | --- | --- | --- | --- | --- | --- | --- |
| Upper Limb |  |  |  |  |  |  |  |  |
| Elbow Flexors | Sling | Pearce et al. 2013 | 40.8(8.8) | 33.0(10.2) ^*^ | % | 21 | -19.12 | -0.91 |
| Wrist Flexors | Brace/Cast | Clark et al. 2008 | ~4.84(0.81) | ~9.19(1.73) | %MEP:Mmax | 7 | 89.88 | 12.84 |
|  |  | Lundbye-Jensen & Nielsen 2008 | 8.7(1.8) | 11.9(2.6) |  | 7 | 36.78 | 5.25 |
| First Dorsal Interosseous | Brace/Cast | Ngomo et al. 2012 | NR | NR | mV | 4 | -54.00 | -13.50 |

^d, days; MEP, motor evoked potential; Mmax, maximal amplitude of Mwave; mV, millivolts; NR, not reported; [ ], multiple pre visits; ~, approximately stated or mean value given.^

Electronic Supplementary Material Table S10 Muscle and Corticospinal Excitability: Changes in compound muscle action potential following immobilisation

| **Muscle group** | **Immobilisation method** | **Study** | **Pre (SD)** | **Post (SD)** | **Units** | **d** | **% Change** | **% Change∙d^-1^** | **Notes** |
| --- | --- | --- | --- | --- | --- | --- | --- | --- | --- |
| **Lower Limb** |  |  |  |  |  |  |  |  |  |
| Plantar Flexors | ULLS | Seynnes et al. 2008, Seynnes et al. 2008 | 5.6(0.6) | 6.7(0.7) ^*^ | mV | 14 | 18.00 ^*^ | 1.29 | Soleus |
|  |  |  | 6.4(0.6) | 6.9(0.5) | mV | 14 | 7.81 | 0.56 | Gastrocnemius |
|  |  | Seynnes et al. 2010 | 8.9(0.5) | 9.3(0.5) | mV | 24 | 4.49 | 0.19 | Resting |
| **Ankle** |  |  |  |  |  |  |  |  |  |
| Plantar Flexors | Brace/ Cast | Lundbye-Jensen & Nielsen 2008 | 32.7  [33.5(12.9), 31.9(12.5)] | 30.9(14) | mV | 14 | -5.50 | -0.39 | Resting, Soleus |
|  |  | Gondin et al. 2004 | 8.09(0.60) | 8.36(0.85) | NR | 14 | 3.34 | 0.24 | Resting, Soleus |
|  |  |  | 7.12(0.99) | 6.81(0.72) | NR | 14 | -4.35 | -0.31 | Gastrocnemius |
| **Upper Limb** |  |  |  |  |  |  |  |  |  |
| Elbow Flexors | Brace/ Cast | Yue et al. 1997 | 9.1(4.3) | 5.0(2.2) | NR | 28 | -45.00 | -3.21 | Resting |
| Wrist Flexors | Brace/ Cast | Clark et al. 2008 | 6.0(1.1) | 5.2(0.9) | mV | 7 | -13.33 | -0.95 |  |
|  |  | Urso et al. 2006 | 0.4(0.05) | ~0.49 ^*^ | NR | 14 | ~23.00 ^*^ | 1.64 | Old |
| Thumb Adduction | Brace/ Cast |  | 0.32(0.04) | ~0.32 | NR | 14 | ~1.00 | 0.07 | Young |
| Thumb Abduction | Brace/ Cast | Sale et al. 1982 | ~9.45(0.92) | ~9.30 (1.29) | mV | 35 | -1.59 | -0.11 | Resting, Group A, n=6 |
|  |  |  | ~9.63(0.41) | ~9.78 (0.44) | mV | 35 | 1.56 | 0.11 | Resting, Group B, n=5 |
| First Dorsal Interosseous | Brace/ Cast | Fuglevand et al. 1995 | 11.4 (4.3) | 9.2(3.4) ^*^ | mV | 7 | -19.00 ^*^ | -2.71 | Resting, n=9 |

^d, days; mV, millivolt; ULLS, unilateral lower limb suspension; NR, not reported; [ ], multiple pre visits; ~, approximately stated or mean value given.^

Electronic Supplementary Material Table S11 Glossary Table

| Measurement | | Description |
| --- | --- | --- |
| Evoked |  |  |
| Muscle Contractility |  |  |
|  | Twitch force | Force developed during an evoked response, usually at rest, in response to peripheral nerve electrical or transcranial magnetic stimulation. |
|  |  |  |
|  | Force development | Time taken to reach maximum force (or a set value) of the evoked twitch response. Measured in time or as a rate of development and suggested to be indicative of calcium release. |
|  |  |  |
|  | Force Relaxation | Time taken to relax to a given level following evoked twitch response. Measured in time or as a rate of relaxation and suggested to be indicative of calcium reuptake. |
|  |  |  |
| Muscle Excitability |  |  |
|  | Mwave amplitude | The amplitude of the compound muscle action potentials in response to the stimulation of a motor nerve. |
|  |  |  |
|  | Hoffman reflex amplitude | The H-reflex is an estimate of alpha motoneuron excitability. |
|  |  |  |
|  | Motor evoked potential amplitude | The amplitude of the compound muscle action potentials in response to the stimulation of the motor cortex |
|  |  |  |
| Central drive |  |  |
|  | Central motor drive | A measure of descending voluntary drive during a contraction. Measured using twitch interpolation technique, central activation ratio or adjusted twitch interpolation. |
| Voluntary |  |  |
|  | Surface EMG | Measurement of the electrical activity produced by skeletal muscle during a contraction. |
|  |  |  |
